# A *Serratia marcescens* PigP Homolog Controls Prodigiosin Biosynthesis, Swarming Motility and Hemolysis and Is Regulated by cAMP-CRP and HexS

**DOI:** 10.1371/journal.pone.0057634

**Published:** 2013-03-01

**Authors:** Robert M. Q. Shanks, Roni M. Lahr, Nicholas A. Stella, Kristin E. Arena, Kimberly M. Brothers, Daniel H. Kwak, Xinyu Liu, Eric J. Kalivoda

**Affiliations:** 1 Charles T. Campbell Laboratory of Ophthalmic Microbiology, Department of Ophthalmology, University of Pittsburgh Eye Center, Pittsburgh, Pennsylvania, United States of America; 2 Department of Chemistry, University of Pittsburgh, Pittsburgh, Pennsylvania, United States of America; University of Birmingham, United Kingdom

## Abstract

Swarming motility and hemolysis are virulence-associated determinants for a wide array of pathogenic bacteria. The broad host-range opportunistic pathogen *Serratia marcescens* produces serratamolide, a small cyclic amino-lipid, that promotes swarming motility and hemolysis. Serratamolide is negatively regulated by the transcription factors HexS and CRP. Positive regulators of serratamolide production are unknown. Similar to serratamolide, the antibiotic pigment, prodigiosin, is regulated by temperature, growth phase, HexS, and CRP. Because of this co-regulation, we tested the hypothesis that a homolog of the PigP transcription factor of the atypical *Serratia* species ATCC 39006, which positively regulates prodigiosin biosynthesis, is also a positive regulator of serratamolide production in *S. marcescens*. Mutation of *pigP* in clinical, environmental, and laboratory strains of *S. marcescens* conferred pleiotropic phenotypes including the loss of swarming motility, hemolysis, and severely reduced prodigiosin and serratamolide synthesis. Transcriptional analysis and electrophoretic mobility shift assays place PigP in a regulatory pathway with upstream regulators CRP and HexS. The data from this study identifies a positive regulator of serratamolide production, describes novel roles for the PigP transcription factor, shows for the first time that PigP directly regulates the pigment biosynthetic operon, and identifies upstream regulators of *pigP*. This study suggests that PigP is important for the ability of *S. marcescens* to compete in the environment.

## Introduction

*Serratia marcescens* is an important opportunistic pathogen of humans [1]. Scores of articles describe hospital outbreaks caused by *S. marcescens*, and clinical studies indicate that it is a major cause of hospital-acquired pneumonia, blood stream infections, surgical site infections, and urinary tract infections [2]–[4]. For example, *S. marcescens* was shown to be the fourth most common cause of early onset pneumonia of intensive care unit (ICU) patients when no antibiotic was administered, and the number one cause of early onset pneumonia of ICU patients to whom systemic antibiotics had been administered [5]. Additionally, *S. marcescens* frequently causes community acquired infections [6] including vision threatening microbial keratitis [7], [8]. Beyond humanity, this gram-negative bacterium is capable of infecting a very wide range of hosts including coral, insects, mammals, nematodes and plants [1]. These varied hosts represent different environmental niches in which *S. marcescens* may compete against other microorganisms.

Swarming motility is a surface-associated group behavior that confers antibiotic resistance [9], [10]. This mode of motility has been noted as a virulence determinant for other gram-negative bacteria such as *Pseudomonas aeruginosa* [11] and *Proteus mirabilis* [12]–[15]. *S. marcescens* flagella and surfactants, known as serrawettins, contribute to swarming motility [16]–[18]. Serratamolide (also known as serrawettin W1), one of these biosurfactants, is a small amino-lipid necessary for swarming in many *S. marcescens* strains, and is co-regulated with the red pigment prodigiosin with respect to temperature and growth phase [19]–[22]. In several organisms, including *S. marcescens*, swarming motility and hemolysis are co-regulated [23]–[29].

Hemolysins are major virulence factors for a wide variety of bacterial pathogens [30]–[32]. A recent study showed that serratamolide, originally characterized for its broad-spectrum antimicrobial activity [33], [34], can be a cytotoxic hemolysin [22]. Biosynthesis of serratamolide is catalyzed by the non-ribosomal peptide synthetase SwrW [20]. Known serratamolide regulators are the LysR family regulator, HexS [21], and the cAMP-receptor protein, CRP [22]. These are both inhibitors of *swrW* transcription, with HexS shown to be a direct inhibitor that binds to the *swrW* promoter [21]. Interestingly, both HexS and CRP also regulate prodigiosin production [21], [35].

Given the importance of serratamolide in swarming motility and hemolysis, we hypothesized the existence of a positive transcriptional regulator(s). Since serratamolide and prodigiosin can be co-regulated, we predicted that positive regulators of prodigiosin would also positively regulate serratamolide production. Relatively little is known about the transcriptional regulators of prodigiosin in *S. marcescens*, with the exception of CRP [35], HexS [21], RssAB [36], and SpnR [37] - all negative regulators. Genetic studies have provided a much more thorough understanding of prodigiosin regulation in *Serratia* species ATCC 39006, an atypical *Serratia* that was isolated in a salt marsh in New Jersey, USA [38]–[44]. Like *S. marcescens*, it produces many secondary metabolites and secreted enzymes that may be of medical and industrial importance [45].

Among the positive regulators of prodigiosin production in *Serratia* sp. ATCC 39006 is PigP, a predicted transcription factor of the XRE family [46]. The PigP protein was reported as a positive regulator of a carbapenem antibiotic and prodigiosin [46]. A *pigP* transposon mutant displayed additional defects in production of cellulase activity, modest reductions in pectinase, and altered swimming motility zones [46]. The use of a transposon with a *lacZ* reporter allowed Fineran and colleagues to show that loss of PigP led to reduced expression of other putative prodigiosin regulators [46]. These include *pigS* and *pigX*, the expression of which were strongly reduced in the *pigP* mutant, as well as *pigQ*, *pigR*, and *rap* whose expression was more modestly reduced. Expression of *pigP* was shown to be independent of quorum sensing, and mutation of *pigP* led to a surprisingly small reduction in *pigA* transcription (∼25–50% reduction in transcript) that manifested in a ∼95% reduction in pigment levels [46]. In the second report regarding PigP, it was shown that mutation of *pigP* did not alter expression of prodigiosin regulator *pigZ* [47]. In the third report that addressed *pigP*, Gristwood and colleagues showed that the absence of PigS, an Ars/SmtB family transcriptional regulator, lead to increased expression of the gene for a pigment inhibitor, *blhA*, and wild-type levels of *blhA* expression could be restored by mutation of the *pigP* gene. However, mutation of *pigP* alone did not significantly alter *blhA* expression [48].

As the study of PigP is relatively new, there are many gaps in knowledge that need to be filled. For example, there are no known upstream regulators of *pigP* expression [45], and it is not known whether PigP can directly regulate transcription of the prodigiosin biosynthetic operon. In this study, we tested the hypothesis that an uncharacterized PigP homolog from *S. marcescens* regulates swarming and hemolysis through serratamolide production. Additionally, it is not clear whether genes identified as secondary metabolite regulators in *Serratia* sp. ATCC 39006 are important in *S. marcescens*. The exception is the *hexS* gene of *S. marcescens* [21], which was named *pigU* in *Serratia* sp. ATCC39006 [46]. Therefore, we tested whether observed phenotypes conferred by *pigP* mutation in *Serratia* sp. ATCC39006 were conserved in *S. marcescens* in addition to our central hypothesis.

Data from the current study indicate a novel role for PigP from environmental, laboratory and clinical strains of *S. marcescens* as a positive regulator of swarming motility and hemolysis through indirect regulation of serratamolide production. *S. marcescens* PigP was found to be a positive regulator of pigment production through direct regulation of the prodigiosin biosynthetic operon, *pigA-N*, as well as being a direct positive regulator of *pigP*. Importantly, upstream regulators for *pigP* were determined; HexS and CRP were observed to be direct and indirect regulators, respectively, of *pigP* transcription.

Like serratamolide, prodigiosin may have a role in competition for environmental niches as it has anti-microbial activity [49]–[51]. Prodigiosin has also been correlated with hydrophobicity-mediated bacterial adhesion that may be important for colonization and distribution of bacteria [52], [53] and has roles in pH and energy homeostasis [54], [55]. The evidence here presents PigP as a key regulator of both an antimicrobial biosurfactant that promotes antibiotic resistance and an antimicrobial pigment, supporting a model where PigP is a key regulator controlling the interplay between *S. marcescens* and other organisms.

## Materials and Methods

### Bacterial Strains, Media, and Growth

Microbial strains used in this study are listed in Table 1. All bacteria were grown with LB (0.5% yeast extract, 1% tryptone, 0.5% NaCl). LB broth was supplemented with adenosine 3′, 5′-cyclic monophosphate (cAMP) where noted; cAMP powder was dissolved in LB and filtered. Antibiotics used to select for plasmids were gentamicin (10 µg/ml) and kanamycin (100 µg/ml). Tetracycline was used at 10 µg/ml to select against *Escherichia coli* in conjugations.

**Table 1:** Strains and plasmids used in this study.

Strain	Description	Reference or source
InvSc1	*Saccharomyces cerevisiae* strain uracil auxotroph	Invitrogen
S17-1 λpir	*Escherichia coli* strain used for conjugation and cloning	[71]
EC100D	*E. coli* strain used for cloning and protein purification	Epicentre
ER2566	*E. coli* strain used for protein purification	New England Biolabs
MZ100	*Staphylococcus aureus* laboratory strain	[58]
K950	*S. aureus* clinical keratitis isolate (MRSA)	[59]
K2315	*Proteus mirabilis* keratitis isolate	Regis Kowalski
CMS376	Serratia marcescens wild type, PIC strain number 3611	Presque Isle Cultures
CMS524	*cyaA-2*– transposon mutation in CMS376	[72]
CMS635	*swrW* – transposon mutation in CMS376	[22]
CMS786	*crp-23*– transposon mutation in CMS376	[72]
CMS836	*pigP-1 (pigP*::pMQ118*)* in CMS376	This study
CMS1033	*SMA3565*::pMQ118 in CMS376	This study
CMS1613	*hexS*::pMQ118 in *swrW* mutant (CMS635)	This study
CMS1687	Δ*crp*, CMS376 with *crp-*Δ*4* allele	[35]
CMS1713	Δ*pigP,* CMS376 with *pigP-*Δ allele	[73]
CMS1742	Δ*crp* Δ*pigP* in CMS376	This study
CMS1744	*hexS*::pMQ118 in Δ*pigP* (CMS1713)	This study
CMS1779	*hexS*::pStvZ3 in WT (CMS376)	This study
CMS1781	*hexS*::pStvZ3 in Δ*pigP* (CMS1713)	This study
CMS1785	*PpigP*-pStvZ3 in WT (CMS376)	This study
CMS2210	Δ*hexS* in WT (CMS376)	[63]
Nima	*S. marcescens* ATCC 29632 laboratory strain	This study
CMS2980	*pigP-1* mutation in Nima	This study
CHASM	Environmental *S. marcescens* isolate	[35]
CMS2981	*pigP*-1 in CHASM	This study
K904	*S. marcescens* clinical keratitis isolate	[35]
CMS2982	*pigP-1* in K904	This study
CMS2234	*swrW*-transposon mutation in *swrW*	This study
K997	*S. marcescens* clinical isolate, non-pigmented	This study
CMS2983	*pigP-1* in K997	This study
CMS3408	*PpigP*-pStvZ3 in Δ*hexS* (CMS2210)	This study
UC1SER	*S. marcescens* isolate from human neonate gut	[64]

### Mutagenesis, Plasmid Construction, and ß-galactosidase Analysis

Chromosomal DNA and plasmid DNA were isolated with commercial kits (Achieve pure DNA cell/tissue, 5 Prime; GenElute Plasmid, Sigma). PCR was performed using a high-fidelity polymerase (Phusion, New England Biolabs). All cloning was performed using yeast *in vivo* cloning [56]. Plasmid details are listed in Table 2.

**Table 2:** Plasmids used in this study.

Plasmid	Description	Reference or source
pMal-C2	Maltose binding protein fusion construct	New England Biolabs
pStvZ3	*ori*R6K *lacZ nptII* promoter probe	[35]
pMQ118	suicide vector *nptII*, *rpsL, oriT, URA3, CEN6/ARSH4*	[73]
pMQ124	*_ori_*ColE1, *_ori_*pRO1600, *aacC1*, *P_BAD_*-*lacZa*, *oriT, URA3, CEN6/ARSH4*	[73]
pMQ125	*_ori_*p15a, *_ori_*pRO1600, *aacC1*, *P_BAD_*-*lacZa*, *oriT, URA3, CEN6/ARSH4*	[73]
pMQ131	*_ori_*pBBR1, *aphA-3*, *P_lac_*-*lacZa*, *oriT, URA3, CEN6/ARSH4*	[73]
pMQ132	*_ori_*pBBR1, *aacC1*, *P_lac_*-*lacZa*, *oriT, URA3, CEN6/ARSH4*	[73]
pMQ179	pMQ118 with internal *pigP* fragment	This study
pMQ196	pMQ118 with internal *hexS* fragment	This study
pMQ200	*_ori_*R6K, *nptII*, *P_BAD_*-*lacZa*, *oriT, URA3, CEN6/ARSH4*	[73]
pMQ212	pMQ125 with the *pigP* open reading frame	This study
pMQ242	pMQ124+ His_8_-CRP	[35]
pMQ248	pMQ131+ P*_flhD_*-*lacZ* (*flhD* promoter)	This study
pMQ253	pStvZ3+ *P_pigP_* (*pigP* promoter)	This study
pMQ268	pStvZ3+ *pigA* internal fragment	[35]
pMQ272	pStvZ3+ *hexS* internal fragment	[22]
pMQ302	pMQ124+ His_9_-*pigP*	This study
pMQ367	pMQ125+ *swrW*	[22]
pMQ368	pMQ200+ *swrW*	This study
pMQ376	*_ori_*pBBR1-based plasmid with *P_swrW_*-tdtomato reporter	[22]
pMQ402	pMAL-C2+ *hexS* (MBP-HexS fusion construct)	[63]

Insertional mutation of the predicted *pigP* homolog (SMA3564), SMA3565, and *hexS*: Internal regions of *pigP*, SMA3565 and *hexS* open reading frames (ORF) were amplified and cloned in the suicide-vector pMQ118 [57]. Primer pairs 1238–1239, 1479–1480, and 1337–1338 respectively, were used to amplify and clone the internal region of *pigP*, SMA3565, and *hexS*. Primers are listed in Table S1. The *pigP* and SMA3565 constructs were introduced into *S. marcescens* as previously described [57]. Briefly, pMQ118 with internal fragments recombine with the respective chromosomal gene yielding a disruption of the gene. In *pigP-1,* the pMQ118 insertion is at base pair 466 out of 615 base pairs ORF. For SMA3565, pMQ118 inserts after base pair 525 out of 897 base pairs for the entire ORF. The *hexS* insertion construct integrates at base pair 400 out of 945. Mutations were verified using PCR. All insertional mutations generated in this manner were grown in kanamycin (100 µg/ml) to maintain the mutation. Controls were performed using CMS376 (wild-type strain) with a kanamycin resistance marker bearing plasmid to ensure that antibiotics alone did not affect the studied phenotypes (data not shown).

The *pigP-lacZ* transcriptional reporter was generated using the pStvZ3 plasmid as previously described [35], using primers 1444 and 1445. Briefly, a 491 base pair promoter region immediately upstream of the *pigP* ORF was amplified and cloned upstream of *lacZ* in pStvZ3 to generate a transcriptional fusion, resulting in plasmid pMQ253. Integration of pMQ253 creates a transcriptional *lacZ* fusion with the native promoter of *pigP*, and places the wild-type *pigP* gene under transcriptional control of the 491 base pair region upstream of *pigP*.

The pMQ248 plasmid has the *flhD* promoter driving expression of *lacZ*, and was used here as a source of *flhD* promoter DNA for controls in electrophoretic mobility shift assays noted below. The plasmid was generated using yeast homologous recombination in which an *oxyR* promoter (to be published elsewhere) was replaced with the *flhD* promoter in a pMQ131 background. Primers for amplification of the *flhD* promoter are listed in Table S1 as 1851 and 1852.

The full-length *pigP* gene was amplified and cloned into pMQ132 under control of the *E. coli P_lac_* promoter using primers 1645 and 1646. The resulting plasmid, pMQ221, was used for complementation analysis. An inducible *pigP* expression plasmid, pMQ212, was made by amplifying the *pigP* ORF from CMS376 and placing it under control of the *E. coli P_BAD_* promoter in vector pMQ125 using primers 1483–1484.

An N-terminal His_9_-tagged version of *pigP* was generated under control of the *E. coli P_BAD_* and recombined into pMQ124 using primer sets: 2093 and 2094, generating plasmid pMQ302.

Full-length *swrW* was amplified using primers detailed previously [22], and cloned into pMQ200 under control of the *E. coli P_BAD_* promoter, generating plasmid pMQ368.

### Detection of *pigP* in *S. marcescens* Isolates

Bacteria from frozen stocks of ocular clinical isolates obtained from the Charles T. Campbell Laboratory of Ophthalmic Microbiology or other strains listed in Table 1 were streaked to single colonies on LB or TSA blood agar plates. DNA was extracted from a single colony using Quick Extract (Epicentre) according to the manufacturers specifications. PCR was performed using standard Taq polymerase (New England Biolabs), and standard conditions using the following primer sets to detect the *pigP* gene (1230–1231 and 1238–1239). *S. marcescens* (CMS376) and *Staphylococcus aureus* (MZ100 and K950) [58], [59] or *Proteus mirabilis* (K2315) chromosomal DNA were used as positive and negative controls respectively. As an additional control for false positive PCR amplicons, the amplified DNA from five randomly chosen isolates was sequenced and all were *pigP* amplicons. Analysis was performed twice with each primer set and any reproducibly generated amplicon of the expected size for any strain was considered a positive result. A quality control PCR reaction was also performed on each DNA preparation to eliminate false negative results using previously described primers, 736– 737, that amplify the *oxyR* gene [57].

### Prodigiosin Production Assays

Single colonies were inoculated in 5 ml of LB medium ± antibiotics and incubated for 18–20 hours (h) on a rotary shaker (TC-7, New Brunswick) at speed setting “8”, (62 rpm). Prodigiosin was extracted from bacterial cells using acidified ethanol, and levels were determined by measuring absorbance at 534 nm, based upon the method of Slater, *et. al*. [60]. Absorbances of extracted prodigiosin and turbidity (OD_600_ nm) of the original culture were read with a spectrophotometer (Molecular Devices, Spectramax Plus) using 1 cm^2^ cuvettes, and the ratio was determined.

### Transcriptional Analysis

ß-galactosidase (ß-gal) assays: after growth of bacterial cultures in LB medium at 30°C to a desired optical density, culture aliquots were pelleted, washed with Z-buffer, and analyzed for ß-gal activity [61]. Lysates were prepared by sonication in Z-buffer and were clarified by centrifugation at 16,100×g for 10 minutes (m). Protein concentration was determined by Bradford analysis, and the same amount of protein from each sample in a given experiment was added to microtiter plate wells and the volume was adjusted to 100 µl with Z-buffer. ONPG (25 µl at 0.2 mg/ml) was added as a substrate, and A_410_ readings were taken with a plate reader after incubation for 10–30 m (Biotek, Synergy 2). Activity was expressed as A_410_/(mg protein×minutes).

Tdtomato fluorescence as a reporter for *swrW* promoter activity was measured from 0.15 ml aliquots of bacterial cultures grown in LB broth with kanamycin using a Synergy 2 plate reader as previously described [22]. The excitation filter for fluorescence was 545/40 nm, the emission filter used measured fluorescence at 590/20 nm. Background fluorescence was equivalent in both strains and the fluorescence was normalized to culture optical density, measured at 600 nm. The experiment was repeated on two different days with similar results.

RNA and cDNA preparation and reverse transcriptase–PCR (RT-PCR) was performed as previously described [22]. The methodology for semi-quantitative RT-PCR and analysis detailed by Marone and colleagues was followed [62]. Primers sequences 2638 and 2639 to detect 16S rDNA were taken from Lin, *et al*., [28]. Primers 1230 and 1231 were used to detect *pigP*. Primers 2911 and 2912 were used to detect *pigA*. Primers 2917–2918 were used to detect *swrW*. A no RT control was performed for every RNA sample and was used to ensure the absence of contaminating chromosomal DNA in cDNA samples (data not shown). Experiments were performed at least three times with two or more independent RNA preparations.

Operon analysis was performed by generating cDNA from RNA from wild-type (WT) cultures harvested at OD_600_ = 2.0 and converted to cDNA with Superscript III reverse transcriptase (Invitrogen) or without RT as a control for chromosomal DNA contamination. Primers to amplify an internal region of *pigP* were 1238 and 1239. Primers to amplify between *pigP* and SMA3565 were 2701 and 2702. Primers to amplify between SMA3565–3566 were 2705 and 2706.

Protein purification and electrophoretic mobility shift assays (EMSA) were performed as previously described using the same reagents [35]. Recombinant His_8_-CRP, expressed from pMQ242, was previously purified [35]. Recombinant His_9_-PigP was purified by nickel-affinity chromatography. Briefly, cultures of EC100D containing empty vector pMQ124 or the His_9_-*pigP* expression vector, pMQ302 were grown overnight in LB medium with gentamicin. Bacteria were diluted to OD_600_ = 0.1 in LB medium with gentamicin and grown with aeration at 30°C until cultures reached OD_600_ = 0.5, at which point L-arabinose (10 mM) was added and cultures were grown for 3 h. Cells were pelleted, washed, and suspended in lysis buffer: sodium phosphate buffer (50 mM), NaCl (300 mM), imidazole (10 mM), triton X-100 (0.1%), pH 8. Clarified lysates were loaded onto columns with HisPur cobalt resin (Pierce), washed twice with wash buffer (same as lysis buffer, but with imidazole at 20 mM). Protein was eluted with elution buffer (as wash buffer without triton X-100, and with imidazole at 100–500 mM). Protein concentration was determined by Bradford analysis. Protein purity was assessed by PAGE analysis where there were no additional bands in the His_9_-PigP eluted fractions, and no purified band in the negative control purification (data not shown).

MBP-HexS and MBP were both purified using maltose-agarose according to the manufacturers specifications (pMAL Protein Fusion and Purification System, New England Biolabs). ER2566 bearing pMAL-C2 for purification of MBP, or pMQ402 [63] for purification of MBP-HexS, grown overnight, subcultured with aeration at 30°C until cultures reached OD_600_ = 0.5, induced with IPTG (0.3 mM) for 3 h, pelleted and frozen. Pellets were washed and suspended in column buffer [Tris-HCl (20 mM), NaCl (200 mM), EDTA (1 mM)], lysed by sonication, clarified by centrifugation, and lysates were loaded onto columns with amylose resin. The resin was washed with 12 volumes of column buffer, and eluted with column buffer containing maltose (10 mM). Protein purity was assessed by PAGE analysis and was greater than 80% pure when analyzed by ImageJ (data not shown).

Labeled DNA amplicons were made with 5′-biotinylated oligonucleotide primers (Integrated DNA Technologies), gel purified and verified by sequencing. A commercial EMSA kit was employed as specified by the manufacturer (Lightshift Chemiluminescent EMSA kit, Pierce) using biotinylated target DNA (1–3 ng), purified His_8_-tagged CRP (≥50 ng), poly-dIdC (500 ng), cAMP (500 µM), and non-labeled competitor DNA (500 ng) in a 20 µl reaction. A 10 µl aliquot of the reaction was separated on a 5% PAGE, TBE gel (Bio-Rad) with running buffer containing 500 µM cAMP. His_9_-PigP and MBP-HexS EMSAs were performed as above, except using poly-dIdC at 1 µg, MgCl_2_ (5 mM), NP-40 (0.05%), 4 ng of biotinylated target DNA, and 500 ng of unlabeled target DNA, 25 µg of MBP-HexS and 33 µg of MBP or 5–20 µg of His_9_-PigP as indicated. EMSA experiments were performed at least three times on different days with similar results. Primers for the *pigA* promoter were 1665 and 1713 (Table S1) and WT chromosomal DNA was used as a template to amplify a 635 bp amplicon that includes 426 bp upstream of the *pigA* start codon as previously described [35]. Primers for the *pigP* promoter were 1346 and 1883 and were used with pMQ253 as a template to amplify a 650 bp amplicon including 492 bp upstream of the *pigP* start codon. Primers for the *swrW* promoter were 2737 and 2738 using WT chromosomal DNA as a template to amplify a 379 bp amplicon including 352 bp of DNA upstream of the *swrW* start codon. Primers for the *flhD* promoter were 1671 and 1672 and pMQ248 was used as a template to amplify a 288 bp amplicon of DNA upstream of the *flhDC* operon as previously described [35]. Primers for the *oxyR* promoter were 1673 and 1675 and used WT chromosomal DNA as a template to amplify a 248 bp amplicon upstream of the *oxyR* gene. Primers to amplify a 345 bp amplicon upstream of the *hexS* gene from WT chromosomal DNA were 2781 and 2782. Primers to amplify a 223 bp amplicon including 199 bp upstream of the *pswP* gene were 2926 and 2928.

Chromatin affinity purification (ChAP) assays were performed as follows. For each strain (WT+pMQ124, WT+pMQ242, and WT+pMQ302), individual single colonies were placed into three, 5 ml cultures consisting of LB medium. These were grown overnight at 30°C with aeration, subcultured in LB medium, grown to OD_600_ =  ∼0.5 at 30°C, supplemented with L-arabinose (13.3 mM), and grown to OD_600_ =  ∼2.0. The three cultures for each group were combined and incubated with formaldehyde (1% final concentration) for 10 m at room temperature. Cross-linking was stopped by the addition of glycine (125 mM) and cells were washed with phosphate-buffered saline (PBS). Cells were resuspended in 1 ml lysis/equilibrium buffer (50 mM sodium phosphate, pH 8, 300 mM NaCl, 10 mM imidazole, and 0.1% triton X-100, 6 µg/ml RNase A), and sonicated for a time and intensity, determined in pilot assays, that sheared most DNA to 500–1000 bp. The lysate was centrifuged at 16,100×g for 10 m at 4°C. DNA was purified from a 100 µl aliquot of the lysate using a Qiagen PCR purification kit and the DNA concentration was determined with a spectrophotometer (Nanodrop N-1000) to normalize input DNA during the affinity purification. A sample was separated on an agarose gel to ensure uniform shearing among samples. A protease inhibitor cocktail (Halt, Pierce Thermo Scientific) was added to the remaining lysate to the manufacturers specifications. An aliquot was kept to represent the total input DNA. Affinity purification was performed on the remaining lysates, normalized by DNA concentration to 100 ng, using nickel-coated paramagnetic beads (PureProteome, Millipore). After two rounds of washing the lysate-incubated beads with lysis/equilibrium buffer, the protein-DNA complexes were separated from the beads using imidazole (1 M) and the eluate was incubated at 65°C for 14–16 h and passed through a Qiagen PCR purification column. The DNA was eluted in the kit-provided elution buffer (50 µl) and PCR was performed on samples using Taq polymerase (New England Biolabs) for 26–32 rounds to ensure amplification in the exponential range [62]. Digital images of non-saturated DNA bands from agarose gels were taken. Primers for amplifying *pigA* were 1665 and 1713, primers for amplifying *pigP* were 1444 and 1445, primers for amplifying *oxyR* were 1432 and 1433.

### Biofilm Formation, Swimming, Swarming and Surfactant Zone Analysis

Static biofilm assays were performed as previously described [57] using polyvinyl chloride as a substrate, and incubation for 20 h at 30°C in LB medium. Biofilms were stained with crystal violet (0.1%); dye was solubilized with glacial acetic acid (33%), and measured spectrophotometrically (A_590_) with a plate reader. Swimming motility was measured with LB agar with 0.3% agar concentration. Surface swarming motility was assessed with LB agar (0.6% agar). Surfactant zones were measured using swarming agar plates. Assays were performed at 30°C. Where noted, serratamolide in DMSO (10 µl of 50 µg/ml) or DMSO (10 µl) were added to the center of swarming agar plates, allowed to dry, and test bacteria were spotted on the center of the plate.

To analyze serratamolide production in the WT and mutant strains of *Serratia marcescens*, 50 ml of overnight cultures were centrifuged. The supernatant was collected and extracted three times with 50 ml of ethyl acetate. The ethyl acetate layer was combined and dried over sodium sulfate and evaporated *in vacuo*. The resulting crude residue was re-dissolved in methanol and analyzed via Shimadzu LCMS-2020 using DIONEX Acclaim 120® C18 column (3 µm particle size, 120 Å pore size, 2.1 × 150 mm dimensions). The mobile phase gradient used for this analysis was as follows: 40% AcCN/60% H_2_O (0 min), 40% AcCN/60% H_2_O (1 min), 90% AcCN/10% H_2_O (15 min), 90% AcCN/10% H_2_O (35 min), 40% AcCN/60% H_2_O (40 min), 40% AcCN/60% H_2_O (45 min). The column oven temperature was set at 40°C and the flow rate was 0.2 ml/min. Serratamolide was monitored at m/z = 515 (for [M+H]^+^) using an ESI-MS detector at positive mode. Authentic serratamolide was isolated as previously reported [22] and used as a positive control in this analysis.

### Statistical Analysis

Two-tailed Student’s *t*-tests and one-way ANOVA with Tukey’s pair-wise post-test analysis with significance set at p<0.05 was performed using Graphpad Prism 5 software.

## Results

### The *S. marcescens pigP* Gene is Required for Full Levels of Prodigiosin Production in Different Strain Backgrounds

The putative *pigP* gene from strain CMS376 used in this study was sequenced from plasmids (pMQ212 and pMQ221) containing the *pigP* ORF (GenBank accession number FJ041060). The predicted protein was found to be 69.1% identical to PigP of *Serratia* sp. ATCC 39006 [46] and 100% amino acid identity to the ORF predicted to code for PigP (SMA3564 ORF) from the sequenced *S. marcescens* strain, Db11.

**Figure 1:**
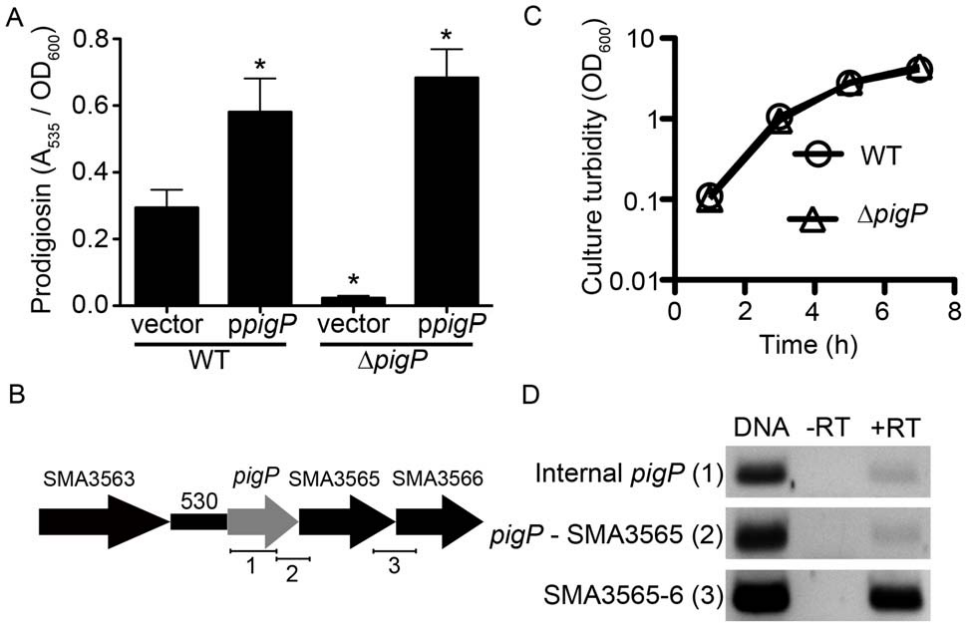
*S. marcescens* PigP positively regulates prodigiosin production. **A.** Complementation analysis of a *pigP* mutant strain. The vector is pMQ132, and p*pigP* refers to pMQ221 (Table 1). Error bars = one standard deviation. * = p<0.05 compared to WT by ANOVA with Tukey’s post-test. **B.** Genetic organization of the chromosome proximal to the *pigP* gene including the predicted *pigP* promoter. Enumerated bars under the genes indicate the regions amplified for operon analysis in panel D. **C.** Growth curve analysis shows similar growth rates for the WT and isogenic *pigP* mutant strains. The average of four biological replicates is shown. **D.** Analysis of the *pigP* operon supports that *pigP* is in a polycistronic message with SMA3565 and SMA3566. RNA isolated from stationary phase cells was treated with reverse transcriptase (+RT) or without reverse transcriptase (−RT) as a negative control. Positive control DNA and experimental samples were assessed with PCR for the presence of amplicons internal to *pigP* as a positive control and that span the genes indicated in the figure. Regions amplified by primers are indicated in by numbered brackets in panel B. Primers for analysis of *pigP*-SMA3565 co-expression extend 144 base pairs into the SMA3565 open frame; those for SMA3565-SMA3566 extend 407 base pairs into the SMA3566 open reading frame.

To test the hypothesis that an *S. marcescens* PigP homolog controls prodigiosin production and promotes swarming and hemolysis, mutations in the *pigP* gene were generated. Because of the inherent artifacts associated with deletion mutations, i.e. deletion of cis-acting regulatory elements for adjacent genes, and with insertion mutations, where polar effects are likely, both types of mutation were used to assess PigP function. Mutant strains of a pigmented laboratory wild-type (WT) strain, CMS376, were generated. One had an insertion mutation in *pigP* (*pigP-1* allele, strain CMS836) and another had an in-frame chromosomal deletion of the *pigP* (*pigP-*Δ allele, strain CMS1713, notated here as Δ*pigP*) in which the first 593 base pairs out of the 615 base pair ORF were deleted (Table 1). A severe defect in pigmentation was measured in both mutant strains (Table 3, 4, Figure 1A, Figure S1A). Both *pigP* mutant strains also exhibited a striking reduction in swarming motility and hemolysis (described below).

**Table 3:** Prodigiosin production in various genetic backgrounds.

Strain^a^	Prodigiosin^b^
WT (CMS376)	0.17±0.04
*pigP* (CMS1713)	0.02±0.01
SMA3565::pMQ118 (CMS1033)	0.20±0.10
*crp* (CMS1687)	1.61±0.26
*crp pigP* (CMS1742)	0.11±0.01
*hexS* (CMS2210)	1.29±0.07
*hexS pigP* (CMS1744)	1.11±0.16

a Strain numbers are defined in Table 1; all are isogenic to WT (CMS376).

b A_534_/OD_600_, measured at 16–18 h, mean of n≥6 independent biological replicates per data point ± one standard deviation.

**Table 4:** Effect of *pigP* insertional mutation on prodigiosin production by different *S. marcescens* isolates.

Strain^a^	Prodigiosin^b^
WT (CMS376)	0.11±0.02
WT *pigP-1* (CMS826)	0.02±0.01
CHASM	0.10±0.01
CHASM *pigP-1* (CMS2981)	<0.01
Nima	0.76±0.11
Nima *pigP-1* (CMS2980)	0.26±0.07
K904	0.63±0.05
K904 *pigP-1* (CMS2982)	0.02±0.02

a Strain numbers are defined in Table 1.

b A_534_/OD_600_, measured at 16–18 h, mean of n≥6 independent biological replicates per data point ± one standard deviation.

The *pigP* gene is the first gene in a predicted operon with three ORFs (Figure 1B); therefore, the prodigiosin defect could originate from a polar effect on the subsequent genes or from the absence of *pigP*. The *pigP* ORF was cloned under control of the *E. coli P_lac_* promoter on a medium copy plasmid (pMQ221) and introduced into the WT and Δ*pigP* strains. A significant increase in prodigiosin production was observed in both the WT and Δ*pigP* strains expressing *pigP* in trans (pMQ221), compared to the vector alone supporting that PigP positively regulates prodigiosin production (p<0.05, ANOVA with Tukey’s Post-test) (Figure 1A). The Δ*pigP* mutant with the vector alone made significantly less prodigiosin (p<0.05, ANOVA with Tukey’s post test) than the WT with the empty vector (8.4±3.7% of WT levels). Importantly, the Δ*pigP* mutant phenotype could be complemented by the ORF *pigP* on a plasmid (pMQ221) (Figure 1A). The pigment phenotype conferred by insertional mutation of *pigP* was also complemented by the intact *pigP* gene on a plasmid (Figure S1A–B, and data not shown). Furthermore, mutation of the subsequent uncharacterized ORF (SMA3565, Figure 1B) did not result in a reduction in prodigiosin (Table 3). Together, these data support that the *pigP* mutant pigment phenotype is due to lack of PigP rather than a polar effect, and that PigP has a positive role in biosynthesis of the secondary metabolite, prodigiosin, similar to what was observed with *Serratia sp.* ATCC 39006 [46].

To determine whether the reduced pigment production was a result of reduced growth, we analyzed growth and recorded identical growth curves for the WT and Δ*pigP* strains under the same conditions used to analyze secondary metabolites (Figure 1C), as was observed with *Serratia sp.* ATCC 39006 [46]. This suggests that there is an active role for PigP in promoting prodigiosin production, rather than an indirect effect associated with a reduced growth rate.

### Analysis of the *pigP* Operon

**Figure 2:**
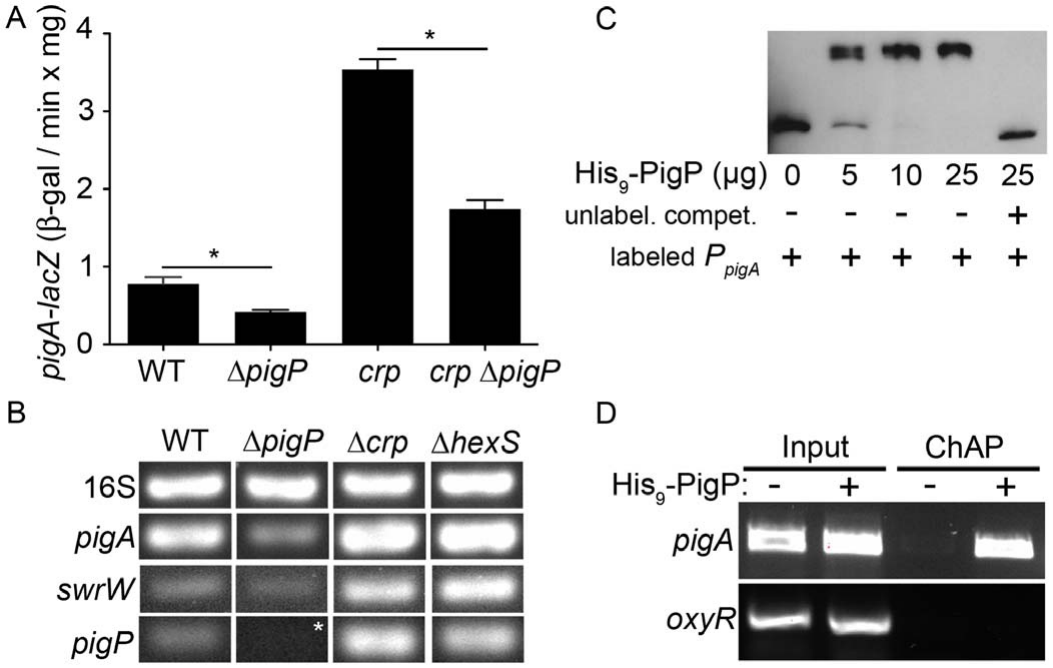
PigP transcriptional regulation of the pigment biosynthetic operon. **A.** Expression of the *pigA* promoter measured using a chromosomal *lacZ* transcriptional fusion at early stationary phase. The average of 4 biological replicates is shown. Error bars indicate one standard deviation. One asterisk indicates a significant difference from (p<0.05, ANOVA Tukey’s post-test). **B.** RT-PCR of cDNA from stationary phase cells (OD_600_ = 3.5) with the 16S target as a control to show equal input cDNA. Genotypes are listed from left to right, and target cDNAs are listed from top to bottom, with 16S rDNA serving as an internal loading control. Representative images are shown. Asterisk indicates that there is no signal here because the *pigP* gene is deleted in this strain; this experiment serves as a negative control. **C.** EMSA assay with biotinylated *pigA* promoter DNA (4 ng) with or without recombinant PigP protein (His_9_-PigP) and with (+) or without (−) unlabeled competitor *pigA* promoter DNA (500 ng). **D.** Chromatin affinity purification (ChAP) enrichment of *pigA* promoter DNA, but not *oxyR* promoter DNA in cells expressing a functional His_9_-PigP (+), but not the vector alone negative control (−). “Input” indicates sheared DNA before affinity purification and shows similar levels of starting DNA.

**Figure 3:**
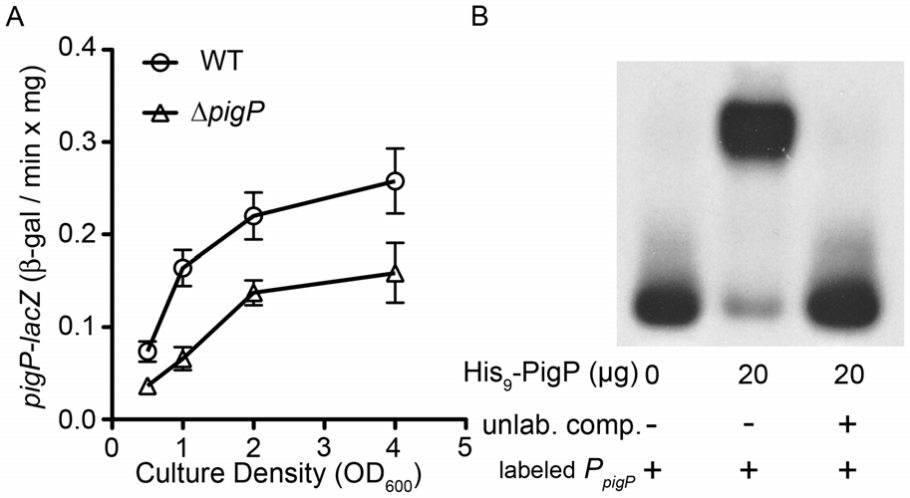
Direct regulation of *pigP* expression by PigP. A. Expression of a chromosomal *pigP*-lacZ transcriptional reporter shows reduced expression in the Δ*pigP* mutant strain (n = 6 biological replicates per time point). **B.** EMSA assay with biotinylated *pigP* promoter DNA (2 ng) as a target that had been incubated with or without recombinant PigP protein (His_9_-PigP) and with (+) or without (−) unlabeled competitor *pigP* promoter DNA (500 ng).

As noted above, the *pigP* gene is in a predicted operon with SMA3565-SMA3566 based on the alignment and proximity of open reading frames and Softberry FGENESB operon prediction software (http://linux1.softberry.com) (Figure 1B), however, this has not been previously studied. To determine whether these genes are in a polycistronic message, we prepared DNase-treated RNA, converted it to cDNA with reverse transcriptase, and tested for co-transcribed messages using PCR (Figure 1D). Primers internal to *pigP* (Figure 1B - 1) were able to direct amplification from chromosomal DNA and reverse transcriptase treated RNA (+RT), but not from no-reverse transcriptase control RNA samples (−RT) indicating that there was undetectable chromosomal DNA contamination (Figure 1D). We reproducibly observed a faint amplicon when using primers that bridge *pigP* and SMA3565 (Figure 1B - 2) and an amplicon bridging both SMA3565 and SMA3566 (Figure 1B - 3) suggesting that these two ORFs are co-transcribed (Figure 1D). The stronger band between SMA3565 to SMA3566 could indicate the presence of another promoter independent of *pigP* expression.

### *S. marcescens* PigP Mediates Prodigiosin Production and the *pigP* gene is Found in Environmental, Clinical and Laboratory Isolates

To determine whether its role in pigment regulation was strain specific, the *pigP* gene was mutated in other pigmented strains by integration of pMQ118 (pMQ179) into the *pigP* ORF of laboratory strain Nima, environmental isolate CHASM, and clinical keratitis isolate K904. We observed a clear decrease in prodigiosin production in all three strains (Table 4) indicating that the role of PigP in prodigiosin regulation is not specific to strain CMS376. Complementation analysis using *pigP* on a plasmid restored pigment to these mutant strains supporting that mutation of *pigP* and not unknown mutations elsewhere in the chromosome were responsible for the observed phenotype (Figure S1B and data not shown).

To determine whether *pigP* was present in clinical strains, we tested a library of 51 pigmentless and 4 pigmented human keratitis isolates and contact lens case contaminants from the Charles T. Campbell laboratory of Ophthalmic Microbiology and one isolate, UC1SER, from a human neonate colon [64] for the presence of the *pigP* gene using PCR. All 56 strains tested exhibited PCR amplicons consistent with the *pigP* gene, a subset are shown in Figure S2, indicating that the gene is conserved among isolates from a variety of niches.

### PigP Directly Regulates Transcription of the Pigment Biosynthetic Operon

To further characterize the role of PigP in prodigiosin biosynthesis, we measured *pigA* transcription, the first gene of the prodigiosinbiosynthetic operon. Transcription of a chromosomal *pigA-lacZ* reporter was significantly reduced in the Δ*pigP* mutant (CMS1713) compared to WT (CMS376) (Figure 2A). This reduction in *pigA* RNA was confirmed using semi-quantitative RT-PCR analysis (Figure 2B). These results show a positive role for PigP in regulation of *S. marcescens* pigment biosynthesis. It is not known whether PigP directly regulates the prodigiosin biosynthesis operon for any organism. To determine whether the positive role in *pigA* transcription is direct or indirect, recombinant poly-histidine tagged PigP (His_9_-PigP) was used in an EMSA assay with *pigA* promoter DNA.

EMSA experiments showed that His_9_-PigP could reproducibly bind to biotin-labeled *pigA* promoter DNA, and the interaction could be inhibited by an excess of unlabeled *pigA* promoter DNA (Figure 2C). As a negative control, His_9_-PigP was unable to bind to the *oxyR* promoter DNA in an EMSA reaction performed under the same conditions (data not shown). To test whether PigP interacts with the *pigA* promoter *in vivo*, a chromatin affinity purification assay (ChAP) was performed, and *pigA* promoter DNA was reproducibly enriched in affinity purified *S. marcescens* cellular fractions from cells expressing His_9_-PigP (Figure 2D, ChAP “+ His_9_-PigP”), but not from fractions with an empty vector control (Figure 2D, ChAP “- His_9_-PigP”). As a negative control, *oxyR* promoter and *fimC*-internal DNA amplicons were not enriched in His_9_-PigP affinity purified fractions (Figure 2D, and data not shown). Together, these results suggest that PigP directly regulates expression of the *pigA-N* biosynthetic operon.

### PigP Positively Regulates *pigP*

It is not known whether PigP regulates expression of the *pigP* promoter. A *lacZ*-transcriptional fusion to the *pigP* promoter was devised to test *pigP* expression in the WT (CMS376) and Δ*pigP* (CMS1713) background. We observed a rapid increase in *pigP* expression between mid- and late- exponential phase in the WT strain and that overall ß-galactosidase levels were up to a maximum difference of 3-fold lower in the Δ*pigP* strain (Figure 3A). Reproducible EMSA assays indicate that His_9_-PigP associates with the *pigP* promoter but not negative controls (*oxyR* promoter) (Figure 3B and data not shown). Together, these data suggest a direct and positive role for PigP in regulation of the *S. marcescens pigP* promoter, suggesting that PigP may directly or indirectly detect the secondary metabolites that it regulates.

### PigP is Necessary for the Hyper-pigmented Phenotype of *crp* Mutants

We previously showed that cAMP-CRP negatively regulates prodigiosin production, but cAMP-CRP did not directly bind to the *pigA* promoter [35]. We performed an epistasis experiment to test the hypothesis that CRP regulates pigment production through PigP. The double *crp pigP* mutant (CMS1742) exhibited prodigiosin production levels similar to the WT (CMS376), but more than the *pigP* mutant (CMS1713) (Table 3).

Using a *pigA-lacZ* reporter construct, we measured the impact of *pigP* mutation on transcription of the prodigiosin biosynthetic operon in a *crp* mutant strain (CMS1687). Similar to previously published results [35], mutation of *crp* confers an increase in *pigA* transcription (Figure 2A–B). The elevated *pigA-lacZ* expression in the *crp* mutant was partially suppressed in the *crp pigP* double mutant (CMS1742) (Figure 2A). These results suggest a regulatory relationship between CRP and PigP, where *pigA* transcriptional control by cAMP-CRP occurs largely, but not completely through PigP. An alternative model is that PigP and CRP independently regulate *pigA* transcription.

### CRP Mediates *pigP* Expression

**Figure 4:**
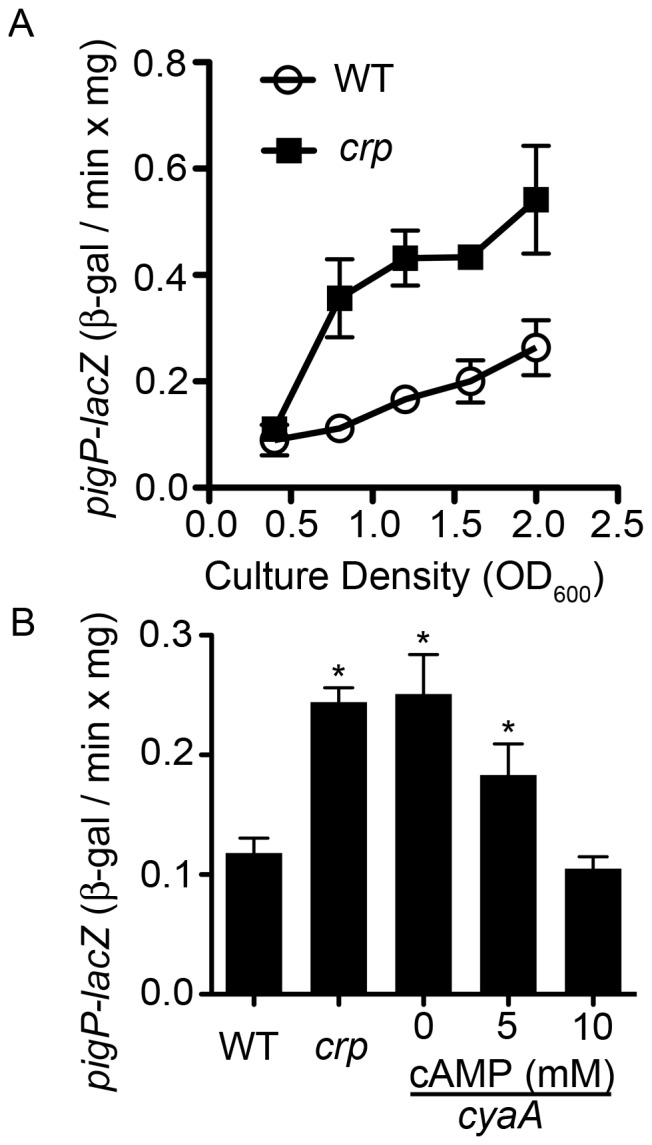
Impact of cAMP-CRP on *pigP* transcription. **A.** ß-galactosidase activity as expressed from the chromosomal *pigP* promoter as a function of culture density. This representative experiment shows the average of 3 biological replicates per genotype. **B.** ß-galactosidase activity from a chromosomal *pigP* reporter in early stationary phase. WT and *crp* strains were grown without exogenous cAMP. The isogenic *cyaA* mutant was grown with 0, 5, or 10 mM cAMP dissolved in the growth medium. This experiment shows the average of 6 biological replicates per cAMP concentration, performed on two different days. Asterisk = significantly different than WT (p<0.05). In this figure “*crp*” refers to the *crp-23* transposon mutant. Error bars = one standard deviation.

Upstream regulators of *pigP* are unknown [45], [46]. The observation that the *crp* mutant’s hyper-red phenotype could be partially suppressed by mutation of *pigP* suggested that the two genes are in a regulatory pathway; therefore, we tested whether *pigP* was transcriptionally regulated by CRP (Figure 2B, 4A–B). A significant (p<0.05) 3.6-fold increase in *pigP*-promoter-driven ß-galactosidase activity was observed in *crp* mutants relative to WT levels at OD_600_ = 0.8 (Figure 4A), and RT-PCR analysis supports that mutation of *crp* increases *pigP* transcript (Figure 2B). A *cyaA* mutant is expected to behave like a *crp* mutant because CyaA catalyzes synthesis of cAMP that mediates CRP function [35]. Similar to the *crp* mutant, expression of *pigP-lacZ* was higher in a *cyaA* mutant (CMS524) (Figure 4B). Furthermore, exogenous cAMP was able to reduce *pigP* expression in a dose-dependent manner in the *cyaA* mutant (Figure 4B). These results support a model that cAMP-CRP negatively regulates *pigP* transcription either directly or indirectly, and that *crp* and *cyaA* mutants exhibit elevated prodigiosin production partially because of increased *pigP* expression.

Although there were no predicted CRP binding sites directly upstream of the *pigP* ORF in our strain background, gel shift assays (EMSA) were performed to determine whether CRP directly or indirectly interacts with these target genes. We did not find evidence that purified CRP bound to a 491 base pair region upstream of *pigP* under the conditions that we used, although a positive control promoter, *flhDC*, was readily bound under the same array of tested conditions (data not shown). Together these data suggest that CRP indirectly regulates *pigP* transcription.

### PigP is Required for Surface Swarming Motility and Serratamolide Production

**Figure 5:**
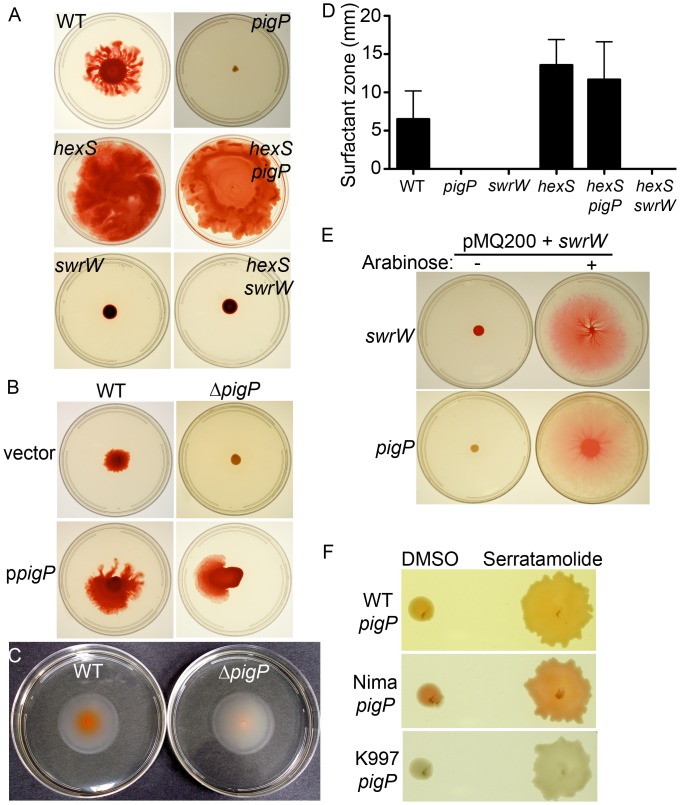
PigP is necessary for swarming motility and serratamolide production, but not swimming motility. **A.** Swarming motility plates show that the *pigP* mutant is defective in surface motility. Mutation of the serratamolide inhibitor *hexS* restores swarming to the *pigP* mutant (*hexS pigP*), and *hexS* requires serratamolide to swarm (*hexS swrW*). **B.** The *pigP* swarming defect can be complemented by the wild-type *pigP* gene provided in trans. Vector refers to pMQ132, and p*pigP* is pMQ221. **C.** Swimming motility plates show similar zones (in all cases “zones” indicates the measurement from the edge of the colony to the outer edge of the observed phenotype in mm) between the WT and *pigP* mutant. **D.** Surfactant zones are absent in strains without *pigP* and the serratamolide biosynthetic gene *swrW*. Mutation of *hexS* restores surfactant zones to the *pigP* mutant. N ≥6 biological replicates per strain. **E.** Arabinose-induced expression of *swrW* is sufficient to restore swarming to a *swrW* and *pigP* mutant. pMQ200+ *swrW* refers to pMQ368. **F.** Purified serratamolide is sufficient to restore swarming to different strain backgrounds with *pigP* mutations, whereas the serratamolide vehicle, DMSO, is not.

Swarming motility over the surface is a virulence associated group behavior [65]–[67]. We observed that the Δ*pigP* strain (CMS1713) was unable to swarm (0%, n = 15, performed on 3 different days), whereas WT was capable of swarming (100%, n = 15) (Figure 5A). The *pigP* mutant swarming deficiency could be complemented by WT *pigP* on a plasmid (Figure 5B).

Factors required for swarming motility include flagella and surface wetting agents, known as serrawettins. Swimming motility assays indicate that the *pigP* mutant (CMS1713) makes functional flagella (Figure 5C). We observed no significant difference (p = 0.14, Student’s *t*-test) in swim zones between the WT (18.7±2.0 mm, n = 11) and Δ*pigP* (19.9±1.65 mm, n = 11) strains.

The surfactant serratamolide (serrawettin W1) generates a visible and transparent halo on the top of swarming agar around the WT strain used in this study [22]. The surfactant zone around the WT culture extended approximately 5 mm beyond the edge of the colony after 24 h, whereas the Δ*pigP* mutant produced no surfactant zone (Figure 5D), and this defect could be complemented by *pigP* on a plasmid (surfactant zones in mm at 18 h: WT+empty vector = 3.7; WT+p*pigP* = 15.2, Δ*pigP*+empty vector = 0, Δ*pigP*+p*pigP* = 13.3). Note that the surfactant zone made by the WT strain was >4-fold larger when *pigP* was expressed on a multicopy plasmid than when the strain had the empty vector (p = 0.02, Student’s *t*-test), suggesting that PigP positively regulates surfactant production. Similar to *pigP*, mutation of the gene coding for SwrW, a non-ribosomal peptide synthetase necessary for production of serratamolide, confers swarming and surfactant zone deficiencies (Figure 5A,D), as has previously been shown [22].

As these data suggest that the Δ*pigP* mutant (CMS1713) does not swarm because it is defective in serratamolide production, we tested whether induced production of serratamolide in a *pigP* mutant could restore swarming motility. Expression of *swrW* from the arabinose-inducible *P_BAD_* promoter (pMQ368) rescued the swarming defect of the Δ*pigP* strain in an arabinose-dependent manner (Figure 5E). Arabinose does not rescue the *pigP* swarming effect without *swrW* on a plasmid (data not shown). Consistent with a lack of serratamolide being the sole reason why *pigP* mutants cannot swarm, the addition of purified serratamolide to Δ*pigP* mutants (50 µg/ml in DMSO) but not by the vehicle control (DMSO) restored swarming motility (Figure 5F).

The swarming defect was also observed when *pigP* was mutated by insertional mutagenesis in the WT strain (CMS376), the laboratory strain Nima, and ocular clinical isolates including a non-pigmented isolate K997 (Figure 5F). These mutants were also restored to swarming by the addition of exogenous serratamolide (Figure 5F).

These data suggest that PigP is an important factor in *swrW* regulation. Semi-quantitative RT-PCR support that there is reproducibly ∼50% more *swrW* transcript in the WT than the isogenic Δ*pigP* mutant in stationary phase cells (OD_600_ = 3.5) (Figure 2B). A previously described *tdtomato*-reporter based *swrW* promoter-probe [22], pMQ376 (Table 2), confirmed that there was less *swrW* expression in the Δ*pigP* mutant (83,476±5117 RFU), compared to the WT (133,221±10670 RFU, p<0.05). EMSA analysis suggests that His_9_-PigP does not directly bind to the *swrW* promoter under a variety of conditions that supported the binding of His_9_-PigP to the *pigP* promoter (Figure S3A), supporting indirect regulation of *swrW* by PigP.

The relatively modest reduction in *swrW* transcript was somewhat surprising given the absence of serratamolide zones around *pigP* mutants on agar plates (Figure 5D), and suggests that growth or media may alter serratamolide production. To test this we measured serratamolide from liquid cultures using liquid chromatograph-mass spectrometry under similar conditions used to assay *swrW* transcript noted above. We observed a reproducible ∼50% reduction in serratamolide in the Δ*pigP* strain compared to the WT, and a complete absence of serratamolide in the *swrW* mutant negative control supernatant (Figure S4).

### PigP Regulates Hemolysis

**Figure 6:**
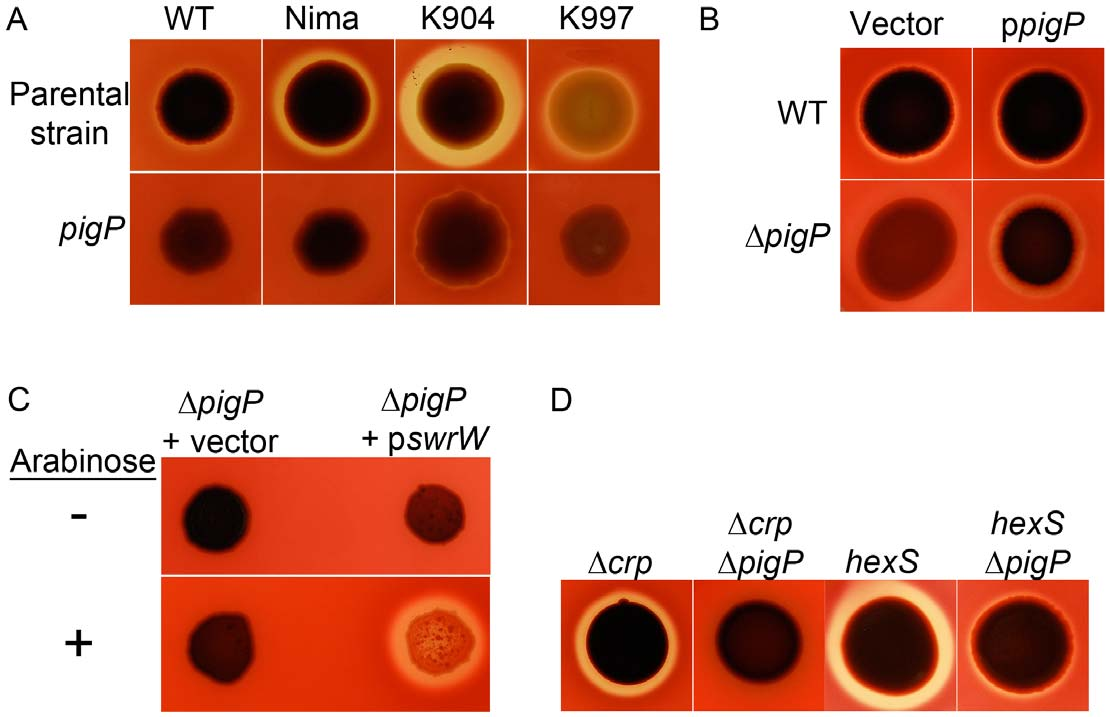
PigP is necessary for hemolysis in laboratory and clinical isolates. A . Hemolytic strains grown on TSA plates with sheep blood show a zone of clearing around colonies indicative of hemolysis. Isogenic *pigP* mutant strains show highly reduced zones of hemolysis. **B.** The hemolysis defect of *pigP* mutants can be complemented by wild-type *pigP* on a plasmid (pMQ221); vector refers to pMQ132. **C.** Arabinose inducible expression of *swrW* is sufficient to restore hemolysis to the *pigP* mutant. The *swrW* gene was expressed from plasmid pMQ367 (p*swrW*), and vector refers to pMQ125. **D.** Mutation of *pigP* reduces the hyper-hemolytic phenotypes of *crp* and *hexS* mutants.

A recent report indicates that serratamolide can be hemolytic [22]. We tested the prediction that *pigP* mutants would be defective in hemolysis. The Δ*pigP* strain (CMS1713) made no zone of clearing on blood agar plates, whereas the WT strain did, and this defect could be complemented by WT *pigP* on a plasmid (Figure 6A–B). A reduction or elimination of secreted hemolytic activity was also observed when *pigP* was disrupted in the WT and Nima laboratory strains, and clinical isolates K904 and K997 (Figure 6A). Arabinose-inducible expression of *swrW* from the Δ*pigP* mutant was able to restore hemolysis to the *pigP* mutant (Figure 6C), supporting that a reduction of *swrW* expression was responsible for the hemolysis defect of *pigP* mutants.

The *hexS* (CMS2210) mutant produces more serratamolide than the WT (Figure 5D) as has been previously shown for a *hexS* mutant in multiple strain backgrounds [20], [22] and for a *crp* mutant in the CMS376 strain background [20], [68] (data not shown), resulting in large zones of clearing on blood agar plates (Fig. 6D). Double mutant analysis shows that the *crp* mutant hyper-hemolysis phenotype is eliminated in the *crp pigP* double mutant (CMS1742), whereas the *pigP hexS* double mutant (CMS1744) has intermediate hemolysis levels (Figure 6D). The WT and *pigP* hemolysis zones were equivalent for the experiment shown in Figure 6D as Figure 6B (data not shown). These data support a model in which PigP and CRP share a common regulatory pathway, where PigP acts downstream of CRP, and HexS and PigP act independently or have a more complex relationship for serratamolide regulation. Together these data support that PigP mediates hemolysis through control of serratamolide biosynthesis.

### Mutation of *hexS* Suppresses the Swarming Deficiency of *pigP* Mutants

It was previously shown that *hexS* mutants produce highly elevated amounts of serratamolide [20], [22]. A Δ*pigP hexS* double mutant was constructed to provide insight into whether HexS and PigP regulate *swrW* through a common pathway. The *hexS* mutant exhibited surfactant zones >2-fold larger than the WT strain (Figure 5D), consistent with its role as a negative regulator of *swrW* expression (Figure 2B). The Δ*pigP hexS* double mutant generated large surfactant zones and a swarming phenotype like the *hexS* mutant (Figure 5A,D). These data indicate that for serratamolide production and swarming, the *hexS* mutation is epistatic to the *pigP* mutation and suggest that HexS acts downstream or independently of PigP. As a control, we generated a *hexS swrW* double mutant that did not produce surfactant zones and was swarming deficient, confirming that serratamolide is required for the *hexS* mutant surfactant zone and swarming phenotypes for the strain used in this study (Figure 5A, D).

Similar to what we observed with swarming, a *hexS* mutation was epistatic to a *pigP* mutation with respect to prodigiosin production (Table 3). Whereas the *pigP* mutant strain (CMS1713) exhibited significantly reduced prodigiosin compared to WT (CMS376), *hexS* (CMS2210) and *pigP hexS* (CMS1744) strains both generated elevated levels of prodigiosin compared to the WT (Table 3).

**Figure 7:**
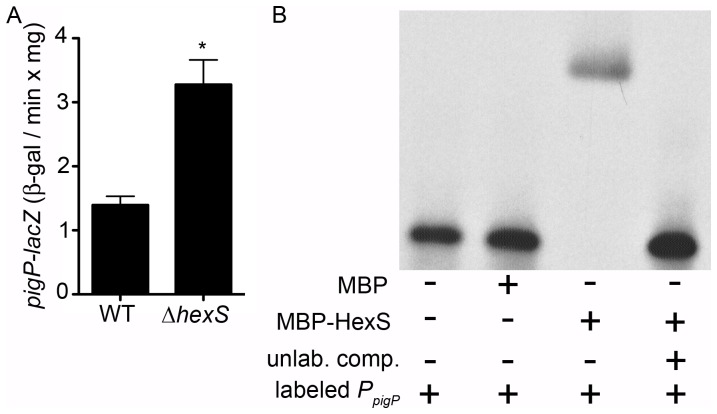
HexS regulates *pigP* expression. **A.** Mutation of *hexS* leads to an increase in output from a chromosomal *pigP* reporter (strains CMS1785 and CMS3408 respectively). Cells were harvested at OD_600_ = 4.0 and ß-gal activity is reported. Data is the mean from 7 biological replicates per genotype. Error bars = one standard-deviation and the asterisk indicates a statistical difference from the WT (p<0.05, Student’s *t*-test). **B.** EMSA analysis indicates that a recombinant maltose binding protein-HexS fusion (MBP-HexS, 25 µg) binds to the labeled *pigP* promoter (2 ng) *in vitro*, whereas the recombinant maltose binding protein (MBP, 33 µg) control does not bind to the *pigP* promoter. Unlabeled *pigP* promoter region competitor DNA (500 ng) was able to inhibit the MBP-HexS induced shift suggesting a specific interaction.

Given the common control of serratamolide and prodigiosin production by both PigP and HexS, we predicted that these two transcription factors are in a regulatory pathway that controls *swrW* expression. It was reported that HexS directly binds to the *swrW* and *pigA* promoters, but not to the promoter of *pswP*, whose gene product is involved in secondary metabolite production [21]. Our data above suggest that PigP does not bind to the *swrW* promoter; therefore, we hypothesized that PigP directly regulates *hexS*. Using a chromosomal *hexS-lacZ* reporter, we measured a modest increase (56±16%) in *hexS* expression measured from over-night stationary phase Δ*pigP* cultures compared to the WT (OD_600_ =  ∼4). However, His_9_-PigP was not observed to bind to the *hexS* promoter using *in vitro* gel shift assays (Figure S3A). Conversely, RT-PCR suggests that HexS is a negative regulator of *pigP* transcription, with elevated *pigP* transcript in the Δ*hexS* mutant (Figure 2B). Similar results were measured with a chromosomal *lacZ* transcriptional reporter for *pigP* expression, where >2-fold more activity was measured in the Δ*hexS* mutant compared to the WT (Figure 7A). EMSA analysis indicates that MBP-HexS can bind to the *pigP* promoter *in vitro* (Figure 7B), similar to positive control promoters, *pigA* and *swrW*, however MBP-HexS did not bind to negative control promoter *pswP* or the *hexS* promoter (Figure S3B). As a control to ensure that the observed shifts were due to the HexS portion of the fusion, MBP alone was included as a control, and failed to elicit the shift of any promoter tested. These data support the model that HexS is a direct negative regulator of PigP.

### The Rugose Colony Phenotype of an Environmental Isolate Requires PigP

**Figure 8:**
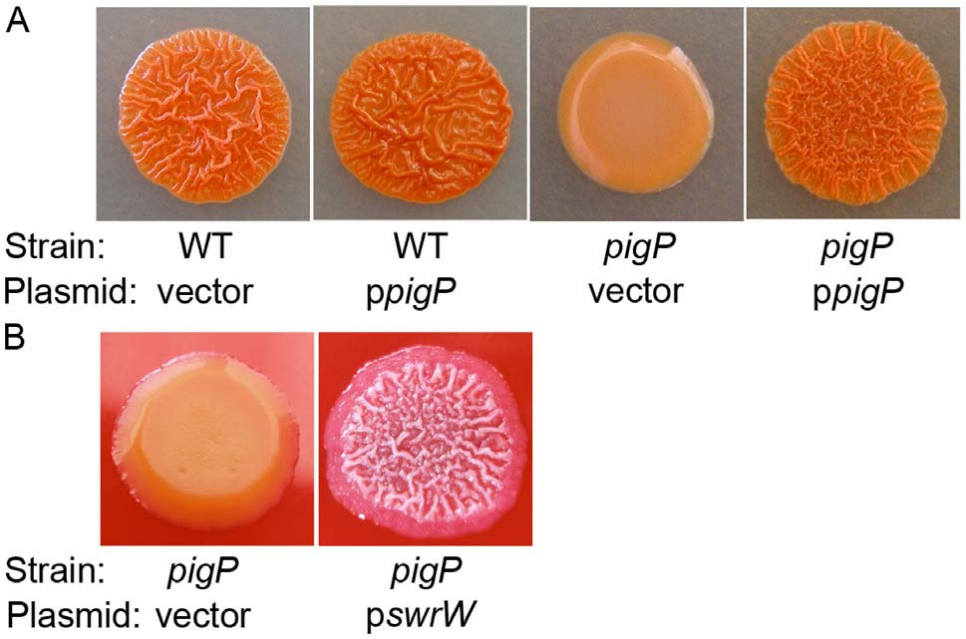
PigP mediates rugose colony morphology. A. The CHASM rugose phenotype is absent in the *pigP* mutant (CMS2981) and can be complemented by wild-type *pigP* on a plasmid (p*pigP* = pMQ221). The vector alone is pMQ132. **B.** The rugose colony morphology defect of the CHASM *pigP* mutant (CMS2981) can be restored through expression of *swrW* from a plasmid (pMQ367), but not from the vector alone (pMQ125).

Another *pigP* mutant phenotype was noted with an environmental isolate of *S. marcescens*, CHASM. We report here that CHASM exhibits a dramatic rugose colony phenotype (Figure 8A). When *pigP* was mutated in CHASM, the colonies changed from rugose and red to smooth and pink (Figure 8A). The rugose phenotype of the CHASM *pigP* mutant was complemented by the wild-type *pigP* gene on a plasmid (Figure 8A). A plasmid with the *swrW* gene (pMQ367) was able to restore the rugose phenotype to the CHASM *pigP* mutant (CMS2982), whereas the vector alone (pMQ125) did not (Figure 8B), suggesting that the rugose phenotype is mediated by serratamolide. At this time the mechanism for serratamolide in this colony morphology phenotype is unknown; however, in other organisms, rugose colony phenotype has been linked to biofilm formation. Static biofilm assays indicate that mutation of *pigP* does not confer a significant biofilm defect under the conditions used (data not shown).

## Discussion

The goal of this study was to determine whether the homolog of a secondary metabolite master regulator protein, PigP, from an atypical environmentally isolated species of *Serratia* positively regulates *S. marcescens* swarming motility and hemolysis and whether it is conserved in prodigiosin regulation. In short, we found that like *Serratia* sp. ATCC 39006, *S. marcescens* uses PigP as a key positive regulator of prodigiosin biosynthesis. Beyond what has been shown in *Serratia* sp. ATCC 39006, we report for the first time that PigP was involved in promoting surfactant production, hemolysis, swarming motility, and a novel rugose colony morphology. Unlike *Serratia* sp. ATCC 39006, mutation of *pigP* in *S. marcescens* did not have a major effect on production of secreted enzymes (Stella and Shanks, unpublished observations), nor upon swimming motility under the tested conditions. One possible mechanism for the observed difference in secreted enzyme production between species is that *Serratia* sp. ATCC 39006 may have a PigP-regulated secretion system not found in *S. marcescens*. This study illustrates that there can be measurable differences in the role of a transcriptional regulator between *Serratia* species.

The swarming defect of *pigP* mutants correlated with a loss of surfactant production and, like the prodigiosin defect, was evident in clinical, environmental and laboratory isolates. Other data suggested that the mechanism for the *pigP* mutant swarming defect is reduced serratamolide production. This model is based on the observations that *pigP* mutants exhibited reduced *swrW* transcript compared to the WT, the swarming defect could be bypassed by inducible expression the *swrW* gene, and that exogenous purified serratamolide could restore swarming to the *pigP* mutant. As serratamolide can act as a hemolysin [22], we tested whether PigP regulated hemolysis. The *pigP* mutant was defective in hemolysis, and inducible expression of *swrW* restored hemolytic zones to the *pigP* mutant. These results suggest that PigP is an important factor in mediating serratamolide production, and therefore swarming motility and hemolysis, by clinical and laboratory strains of *S. marcescens*.

**Figure 9:**
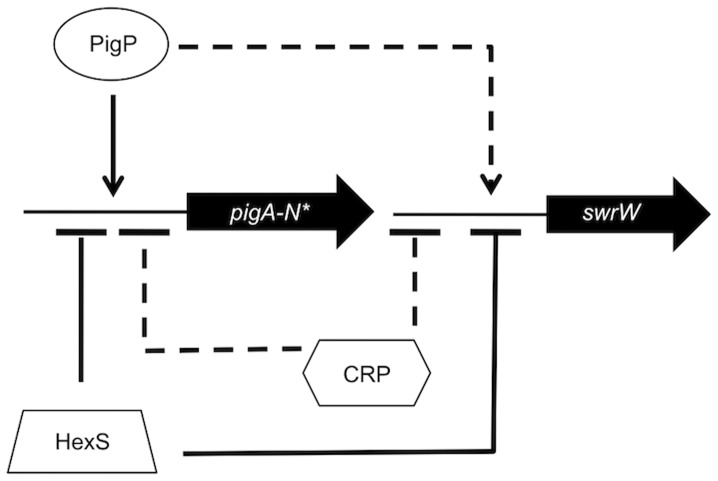
Model for regulation of secondary metabolite biosynthesis genes by the transcription factors described in this study. The secondary metabolite genes (*pigA-N* for prodigiosin and *swrW* for serratamolide) and the *pigP* regulator gene (not shown) are negatively (bar) and directly (solid line) regulated by HexS. They are negatively and indirectly (dashed line) regulated by CRP. The *pigA-N* operon and *pigP* are positively (arrow) and directly regulated by PigP, whereas *swrW* is indirectly regulated by PigP. The asterisk indicates that the same pattern of regulation for the *pigA-N* operon is observed for the *pigP* gene.

While it has been previously shown that mutation of the *pigP* gene leads to altered transcription of a number of genes in *Serratia* sp. ATCC 39006, it has not been shown that the PigP protein directly binds to the promoters that it regulates. Here we provide evidence that the *S. marcescens* PigP homolog directly regulates expression of the prodigiosin biosynthetic operon, *pigA-N*, and the *pigP* gene. Our data indicate that mutation of *pigP* leads to a reduction of *swrW* expression, but recombinant PigP was not shown to bind to the *swrW* promoter *in vitro*. It is possible that the PigP promoter can bind to *swrW in vivo* by itself or in conjunction with another protein, and that the conditions of our EMSA reaction were not appropriate, although a battery of reaction conditions were employed. These data support the model that PigP controls secondary metabolite production directly (*pigA*) and indirectly (*swrW*) through unknown regulator(s) (Figure 9). Consistent with this model, there is evidence that the *Serratia* sp. ATCC 39006 PigP protein regulates a number of other transcriptional regulators involved in secondary metabolite control [45].

It may be noted that some of the changes in gene expression elicited by mutation of *pigP* are not particularly dramatic, e.g. ∼50% reduction in *pigA* transcript. However, this is similar to what has been reported for other regulators that effect prodigiosin and other secondary metabolite production, where modest changes in transcript correlate with large phenotypic changes [36], [46].

It was previously reported that there are no known regulators of *pigP* [45], [46]. Here we provide data that cAMP-CRP is an indirect upstream regulator of *pigP* (Figure 9). The *pigP* gene was found necessary for the hyper-pigment and hyper-hemolysis phenotypes of a *crp* mutant, and increased expression of the *pigP* gene in *crp* and *cyaA* mutants, suggesting that PigP acts downstream of cAMP-CRP to regulate secondary metabolite genes. The absence of cAMP-CRP binding to the *pigP* promoter suggests that there is an intermediate regulators(s). Multiple proteins involved in carbon utilization also contribute to pigment regulation including the transcription factor PigT [38] and components of bacterial electron transport chains [41], [69] underscoring that prodigiosin may be an important factor in control of energy homeostasis [55].

In addition to cAMP-CRP, we present data supporting that HexS is a direct regulator of *pigP* expression (Figure 9). A *hexS pigP* double mutant exhibited a *hexS* mutant-like phenotype with respect to swarming motility and prodigiosin levels but not hemolysis, where an intermediate phenotype was observed. The discordant phenotypes from the same strain in different assays may be due to different thresholds of serratamolide necessary to elicit each phenotype, or differential production of serratamolide under the different experimental conditions used in these assays. Nevertheless, these genetic data imply either a direct relationship with PigP acting upstream of HexS, for which we have little evidence (mutation of *pigP* had little effect on *hexS* expression and PigP did not bind to the *hexS* promoter), or through an independent relationship in which HexS and PigP can both independently regulate target genes. This second model is supported by evidence that both PigP and HexS regulate and bind to *swrW* and *pigA* promoters. Our transcriptional and EMSA data support the model that HexS can further influence the pathway by directly regulating *pigP* expression. Together supporting the model that HexS can influence secondary metabolite production both directly through control of *pigA-N* and *swrW* and indirectly as a direct upstream regulator of *pigP*.

Interestingly, we observed that PigP was able to directly regulate expression of its own promoter in a positive manner suggesting that it may directly or indirectly respond to the secondary metabolites that it regulates. Future studies will be focused on determining the binding site of HexS and PigP and elucidating the mechanism by which these opposing regulators mediate transcription of the prodigiosin biosynthetic operon and determining the environmental stimuli that these regulators respond to.

PigP regulates carbapenem antibiotic in *Serratia* sp. ATCC 39006 and prodigiosin biosynthesis in both *S. marcescens* and ATCC 39006 [45], [46]. Prodigiosin has antibiotic properties [49] and is excreted from cells in extracellular vesicles [70]. Similarly, serratamolide has antimicrobial properties [33], [34] and is thought to be necessary for the generation of extracellular vesicles [70]. One possible function of PigP is to regulate production of antibiotics to compete for limited nutrients against other organisms, consistent with our observation that PigP is necessary to inhibit the growth of a gram-positive organism adjacent to *S. marcescens* colonies (Shanks, unpublished observations). cAMP-CRP is best known for regulation of metabolic pathways in response to environmental carbon sources, but it also regulates production of adhesins, flagella and other factors involved in interacting with the environment. Therefore, PigP’s control of competitive factors ties in well with the function of cAMP-CRP in adaptation of bacteria to the environment based upon nutrient cues. However, given the number of transcription factors that contribute to secondary metabolism further research is necessary to discover and characterize the other inputs for regulating secondary metabolites in *S. marcescens*.

## Supporting Information

Figure S1 **Complementation of prodigiosin phenotype conferred by insertional mutation of** ***pigP*** **.** A. Photograph of WT (CMS376) or the *pigP* mutant (CMS836) with the vector (pMQ125) or p*pigP* (pMQ212) grown on LB agar supplemented with arabinose (4 mM). B. Prodigiosin production by the environmental isolate, CHASM, and isogenic *pigP*-insertion mutant (CMS2981) being either the empty vector (pMQ132) or p*pigP* (pMQ221) grown in LB medium. The average of six independent biological replicates is shown.
(PDF)

Figure S2 **Amplification of** ***pigP*** **from** ***S. marcescens*** **isolates.** PCR was used to amplify a 138 base pair amplicon from a variety of *S. marcescens* strains. Db11 and CMS376 served as positive controls, whereas *Proteus* and *Staphylococcus* chromosomal DNA served as negative controls. Amplicons were separated on TBE-PAGE gels and stained with ethidium bromide.
(PDF)

Figure S3 **EMSA analysis of His9-PigP and MBP-HexS with promoters of interest.** A) His9-PigP exhibited a gel shift of the *pigP* promoter but not the *swrW* or *hexS* promoters. B) MPB-HexS retarded migration of *pigA* and *swrW* promoters (positive controls), but not the *pswP* promoter (negative control) or the *hexS* promoter. MBP alone did not induce a gel shift of any promoter. Excess unlabeled promoter DNA could compete successfully for MPB-HexS binding to *pigA* and *swrW* promoters. Biotin-labeled promoters (Label. Promoter) were used at 2 ng per reaction and unlabeled promoters (Unlab promoter) were used at 500 ng per reaction.
(PDF)

Figure S4 **Analysis of serratamolide from** ***S. marcescens*** **culture supernatants.** LC-MS was used to measure serratamolide levels in culture supernatants from the WT (CMS376) the Δ*pigP* strain (CMS1713) and the negative control *swrW* mutant (CMS635). Purified serratamolide was used as a positive control. The serratamolide peaks are boxed in red the scale of the trace is shown on the left hand side. A representative experiment is shown.
(PDF)

Table S1 **Primers used in this study.**
(DOCX)

## References

[1] Mahlen SD (2011) *Serratia* infections: from military experiments to current practice. Clin Microbiol Rev 24: 755–791. 21976608 10.1128/CMR.00017-11 PMC3194826

[2] Diekema DJ, Pfaller MA, Jones RN, Doern GV, Winokur PL, et al (1999) Survey of bloodstream infections due to gram-negative bacilli: frequency of occurrence and antimicrobial susceptibility of isolates collected in the United States, Canada, and Latin America for the SENTRY Antimicrobial Surveillance Program, 1997. Clin Infect Dis 29: 595–607. 10530454 10.1086/598640

[3] Richards MJ, Edwards JR, Culver DH, Gaynes RP (2000) Nosocomial infections in combined medical-surgical intensive care units in the United States. Infect Control Hosp Epidemiol 21: 510–515. 10968716 10.1086/501795

[4] Jones RN (2010) Microbial etiologies of hospital-acquired bacterial pneumonia and ventilator-associated bacterial pneumonia. Clin Infect Dis 51 Suppl 1S81–87. 20597676 10.1086/653053

[5] Verhamme KM, De Coster W, De Roo L, De Beenhouwer H, Nollet G, et al (2007) Pathogens in early-onset and late-onset intensive care unit-acquired pneumonia. Infect Control Hosp Epidemiol 28: 389–397. 17385143 10.1086/511702

[6] Laupland KB, Parkins MD, Gregson DB, Church DL, Ross T, et al (2008) Population-based laboratory surveillance for *Serratia* species isolated in a large Canadian health region. Eur J Clin Microbiol Infect Dis 27: 89–95. 17960436 10.1007/s10096-007-0400-7

[7] Hume EB, Conerly LL, Moreau JM, Cannon BM, Engel LS, et al (1999) *Serratia marcescens* keratitis: strain-specific corneal pathogenesis in rabbits. Curr Eye Res 19: 525–532. 10550795 10.1076/ceyr.19.6.525.5283

[8] Hume EB, Willcox MD (2004) Emergence of *Serratia marcescens* as an ocular surface pathogen. Arch Soc Esp Oftalmol 79: 475–477. 15523567

[9] Butler MT, Wang Q, Harshey RM (2010) Cell density and mobility protect swarming bacteria against antibiotics. Proc Natl Acad Sci U S A 107: 3776–3781. 20133590 10.1073/pnas.0910934107 PMC2840483

[10] Kearns DB (2010) A field guide to bacterial swarming motility. Nat Rev Microbiol 8: 634–644. 20694026 10.1038/nrmicro2405 PMC3135019

[11] Overhage J, Bains M, Brazas MD, Hancock RE (2008) Swarming of *Pseudomonas aeruginosa* is a complex adaptation leading to increased production of virulence factors and antibiotic resistance. J Bacteriol 190: 2671–2679. 18245294 10.1128/JB.01659-07 PMC2293252

[12] Allison C, Lai HC, Hughes C (1992) Co-ordinate expression of virulence genes during swarm-cell differentiation and population migration of *Proteus mirabilis* . Mol Microbiol 6: 1583–1591. 1495387 10.1111/j.1365-2958.1992.tb00883.x

[13] Fraser GM, Claret L, Furness R, Gupta S, Hughes C (2002) Swarming-coupled expression of the *Proteus mirabilis hpmBA* haemolysin operon. Microbiology 148: 2191–2201. 12101306 10.1099/00221287-148-7-2191 PMC2528290

[14] Walker KE, Moghaddame-Jafari S, Lockatell CV, Johnson D, Belas R (1999) ZapA, the IgA-degrading metalloprotease of *Proteus mirabilis*, is a virulence factor expressed specifically in swarmer cells. Mol Microbiol 32: 825–836. 10361285 10.1046/j.1365-2958.1999.01401.x

[15] Wang WB, Chen IC, Jiang SS, Chen HR, Hsu CY, et al (2008) Role of RppA in the regulation of polymyxin b susceptibility, swarming, and virulence factor expression in *Proteus mirabilis* . Infect Immun 76: 2051–2062. 18316383 10.1128/IAI.01557-07 PMC2346679

[16] Matsuyama T, Sogawa M, Nakagawa Y (1989) Fractal spreading growth of *Serratia marcescens* which produces surface active exolipids. FEMS Microbiol Lett 52: 243–246. 2693193 10.1016/0378-1097(89)90204-8

[17] O’Rear J, Alberti L, Harshey RM (1992) Mutations that impair swarming motility in *Serratia marcescens* 274 include but are not limited to those affecting chemotaxis or flagellar function. J Bacteriol 174: 6125–6137. 1400161 10.1128/jb.174.19.6125-6137.1992 PMC207679

[18] Matsuyama T, Bhasin A, Harshey RM (1995) Mutational analysis of flagellum-independent surface spreading of *Serratia marcescens* 274 on a low-agar medium. J Bact 177: 987–991. 7860610 10.1128/jb.177.4.987-991.1995 PMC176693

[19] Sunaga S, Li H, Sato Y, Nakagawa Y, Matsuyama T (2004) Identification and characterization of the *pswP* gene required for the parallel production of prodigiosin and serrawettin W1 in *Serratia marcescens* . Microbiol Immunol 48: 723–728. 15502404 10.1111/j.1348-0421.2004.tb03597.x

[20] Li H, Tanikawa T, Sato Y, Nakagawa Y, Matsuyama T (2005) *Serratia marcescens* gene required for surfactant serrawettin W1 production encodes putative aminolipid synthetase belonging to nonribosomal peptide synthetase family. Microbiol Immunol 49: 303–310. 15840955 10.1111/j.1348-0421.2005.tb03734.x

[21] Tanikawa T, Nakagawa Y, Matsuyama T (2006) Transcriptional downregulator HexS controlling prodigiosin and serrawettin W1 biosynthesis in *Serratia marcescens* . Microbiol Immunol 50: 587–596. 16924143 10.1111/j.1348-0421.2006.tb03833.x

[22] Shanks RM, Stella NA, Lahr RM, Wang S, Veverka TI, et al (2012) Serratamolide is a hemolytic factor produced by *Serratia marcescens* . PLoS One 7: e36398. 22615766 10.1371/journal.pone.0036398 PMC3353980

[23] Allison C, Coleman N, Jones PL, Hughes C (1992) Ability of *Proteus mirabilis* to invade human urothelial cells is coupled to motility and swarming differentiation. Infect Immun 60: 4740–4746. 1398984 10.1128/iai.60.11.4740-4746.1992 PMC258226

[24] Carpinella MC, De Bellis L, Joray MB, Sosa V, Zunino PM, et al (2011) Inhibition of development, swarming differentiation and virulence factors in Proteus mirabilis by an extract of *Lithrea molleoides* and its active principle (Z,Z)-5-(trideca-4′,7′-dienyl)-resorcinol. Phytomedicine 18: 994–997. 21514124 10.1016/j.phymed.2011.03.003

[25] Dacheux D, Goure J, Chabert J, Usson Y, Attree I (2001) Pore-forming activity of type III system-secreted proteins leads to oncosis of *Pseudomonas aeruginosa*-infected macrophages. Mol Microbiol 40: 76–85. 11298277 10.1046/j.1365-2958.2001.02368.x

[26] Givaudan A, Lanois A (2000) flhDC, the flagellar master operon of *Xenorhabdus nematophilus*: requirement for motility, lipolysis, extracellular hemolysis, and full virulence in insects. J Bacteriol 182: 107–115. 10613869 10.1128/jb.182.1.107-115.2000 PMC94246

[27] Hsueh YH, Somers EB, Lereclus D, Ghelardi E, Wong AC (2007) Biosurfactant production and surface translocation are regulated by PlcR in *Bacillus cereus* ATCC 14579 under low-nutrient conditions. Appl Environ Microbiol 73: 7225–7231. 17921286 10.1128/AEM.00690-07 PMC2168185

[28] Lin CS, Horng JT, Yang CH, Tsai YH, Su LH, et al (2010) RssAB-FlhDC-ShlBA as a major pathogenesis pathway in *Serratia marcescens* . Infect Immun 78: 4870–4881. 20713626 10.1128/IAI.00661-10 PMC2976324

[29] Ulrich RL, Hines HB, Parthasarathy N, Jeddeloh JA (2004) Mutational analysis and biochemical characterization of the *Burkholderia thailandensis* DW503 quorum-sensing network. J Bacteriol 186: 4350–4360. 15205437 10.1128/JB.186.13.4350-4360.2004 PMC421622

[30] Bhakdi S, Tranum-Jensen J (1991) Alpha-toxin of *Staphylococcus aureus* . Microbiol Rev 55: 733–751. 1779933 10.1128/mr.55.4.733-751.1991 PMC372845

[31] Braun V, Focareta T (1991) Pore-forming bacterial protein hemolysins (cytolysins). Crit Rev Microbiol 18: 115–158. 1930675 10.3109/10408419109113511

[32] Schnupf P, Portnoy DA (2007) Listeriolysin O: a phagosome-specific lysin. Microbes Infect 9: 1176–1187. 17720603 10.1016/j.micinf.2007.05.005

[33] Dwivedi D, Jansen R, Molinari G, Nimtz M, Johri BN, et al (2008) Antimycobacterial serratamolides and diacyl peptoglucosamine derivatives from Serratia sp. J Nat Prod 71: 637–641. 18303848 10.1021/np7007126

[34] Wasserman HH, Keggi JJ, Mckeon JE (1961) Serratamolide, a metabolic product of *Serratia* . J Am chem Soc 83: 4107–4108.

[35] Kalivoda EJ, Stella NA, Aston MA, Fender JE, Thompson PP, et al (2010) Cyclic AMP negatively regulates prodigiosin production by *Serratia marcescens* . Res Microbiol 161: 158–167. 20045458 10.1016/j.resmic.2009.12.004 PMC2846241

[36] Horng YT, Chang KC, Liu YN, Lai HC, Soo PC (2010) The RssB/RssA two-component system regulates biosynthesis of the tripyrrole antibiotic, prodigiosin, in *Serratia marcescens* . Int J Med Microbiol 300: 304–312. 20347390 10.1016/j.ijmm.2010.01.003

[37] Horng YT, Deng SC, Daykin M, Soo PC, Wei JR, et al (2002) The LuxR family protein SpnR functions as a negative regulator of N-acylhomoserine lactone-dependent quorum sensing in *Serratia marcescens* . Mol Microbiol 45: 1655–1671. 12354232 10.1046/j.1365-2958.2002.03117.x

[38] Fineran PC, Everson L, Slater H, Salmond GP (2005) A GntR family transcriptional regulator (PigT) controls gluconate-mediated repression and defines a new, independent pathway for regulation of the tripyrrole antibiotic, prodigiosin, in Serratia. Microbiology 151: 3833–3845. 16339930 10.1099/mic.0.28251-0

[39] Fineran PC, Williamson NR, Lilley KS, Salmond GP (2007) Virulence and prodigiosin antibiotic biosynthesis in *Serratia* are regulated pleiotropically by the GGDEF/EAL domain protein, PigX. J Bacteriol 189: 7653–7662. 17766413 10.1128/JB.00671-07 PMC2168757

[40] Gristwood T, Fineran PC, Everson L, Williamson NR, Salmond GP (2009) The PhoBR two-component system regulates antibiotic biosynthesis in *Serratia* in response to phosphate. BMC Microbiol 9: 112. 19476633 10.1186/1471-2180-9-112 PMC2695467

[41] McNeil MB, Clulow JS, Wilf NM, Salmond GP, Fineran PC (2012) SdhE is a conserved protein required for flavinylation of succinate dehydrogenase in bacteria. J Biol Chem 287: 18418–18428. 22474332 10.1074/jbc.M111.293803 PMC3365757

[42] Ramsay JP, Salmond GP (2012) Quorum sensing-controlled buoyancy through gas vesicles: Intracellular bacterial microcompartments for environmental adaptation. Commun Integr Biol 5: 96–98. 22482022 10.4161/cib.18532 PMC3291326

[43] Wilf NM, Salmond GP (2012) The stationary phase sigma factor, RpoS, regulates the production of a carbapenem antibiotic, a bioactive prodigiosin and virulence in the enterobacterial pathogen *Serratia sp.* ATCC 39006. Microbiology 158: 648–658. 22194349 10.1099/mic.0.055780-0

[44] Wilf NM, Williamson NR, Ramsay JP, Poulter S, Bandyra KJ, et al (2011) The RNA chaperone, Hfq, controls two luxR-type regulators and plays a key role in pathogenesis and production of antibiotics in *Serratia sp.* ATCC 39006. Environ Microbiol 13: 2649–2666. 21824244 10.1111/j.1462-2920.2011.02532.x

[45] Williamson NR, Fineran PC, Leeper FJ, Salmond GP (2006) The biosynthesis and regulation of bacterial prodiginines. Nat Rev Microbiol 4: 887–899. 17109029 10.1038/nrmicro1531

[46] Fineran PC, Slater H, Everson L, Hughes K, Salmond GP (2005) Biosynthesis of tripyrrole and beta-lactam secondary metabolites in *Serratia*: integration of quorum sensing with multiple new regulatory components in the control of prodigiosin and carbapenem antibiotic production. Mol Microbiol 56: 1495–1517. 15916601 10.1111/j.1365-2958.2005.04660.x

[47] Gristwood T, Fineran PC, Everson L, Salmond GP (2008) PigZ, a TetR/AcrR family repressor, modulates secondary metabolism via the expression of a putative four-component resistance-nodulation-cell-division efflux pump, ZrpADBC, in *Serratia sp.* ATCC 39006. Mol Microbiol 69: 418–435. 18485072 10.1111/j.1365-2958.2008.06291.x

[48] Gristwood T, McNeil MB, Clulow JS, Salmond GP, Fineran PC (2011) PigS and PigP regulate prodigiosin biosynthesis in *Serratia* via differential control of divergent operons, which include predicted transporters of sulfur-containing molecules. J Bacteriol 193: 1076–1085. 21183667 10.1128/JB.00352-10 PMC3067589

[49] Gerber NN (1975) Prodigiosin-like pigments. Crit Rev Microbiol 3: 469–485. 10.3109/10408417509108758 1095305

[50] Vining LC (1990) Functions of secondary metabolites. Annu Rev Microbiol 44. 10.1146/annurev.mi.44.100190.002143 2252388

[51] Williamson NR, Fineran PC, Ogawa W, Woodley LR, Salmond GP (2008) Integrated regulation involving quorum sensing, a two-component system, a GGDEF/EAL domain protein and a post-transcriptional regulator controls swarming and RhlA-dependent surfactant biosynthesis in *Serratia* . Environ Microbiol 10: 1202–1217. 18294208 10.1111/j.1462-2920.2007.01536.x

[52] Rosenberg M, Blumberger Y, Judes H, Bar-Ness R, Rubinstein E, et al (1986) Cell surface hydrophobicity of pigmented and nonpigmented clinical *Serratia marcescens* strains. Infect Immun 51: 932–935. 3512440 10.1128/iai.51.3.932-935.1986 PMC260988

[53] Rosenberg M, Kjelleberg S (1986) Hydrophobic interactions: role in bacterial adhesion. Adv Microbial Ecol 9: 353–393.

[54] Rius N, Sole M, Fancia A, Loren JG (1994) Buffering capacity of pigmented and nonpigmented strains of *Serratia marcescens* . Appl Environ Microbiol 60: 2152–2154. 16349300 10.1128/aem.60.6.2152-2154.1994 PMC201615

[55] Haddix PL, Jones S, Patel P, Burnham S, Knights K, et al (2008) Kinetic analysis of growth rate, ATP, and pigmentation suggests an energy-spilling function for the pigment prodigiosin of *Serratia marcescens* . J Bacteriol 190: 7453–7463. 18805986 10.1128/JB.00909-08 PMC2576671

[56] Shanks RM, Caiazza NC, Hinsa SM, Toutain CM, O’Toole GA (2006) *Saccharomyces cerevisiae*-based molecular tool kit for manipulation of genes from gram-negative bacteria. Appl Environ Microbiol 72: 5027–5036. 16820502 10.1128/AEM.00682-06 PMC1489352

[57] Shanks RM, Stella NA, Kalivoda EJ, Doe MR, O’Dee DM, et al (2007) A *Serratia marcescens* OxyR homolog mediates surface attachment and biofilm formation. J Bacteriol 189: 7262–7272. 17675374 10.1128/JB.00859-07 PMC2168423

[58] Shanks RM, Donegan NP, Graber ML, Buckingham SE, Zegans ME, et al (2005) Heparin stimulates *Staphylococcus aureus* biofilm formation. Infect Immun 73: 4596–4606. 16040971 10.1128/IAI.73.8.4596-4606.2005 PMC1201187

[59] Kowalski RP, Romanowski EG, Mah FS, Shanks RM, Gordon YJ (2010) Topical levofloxacin 1.5% overcomes in vitro resistance in rabbit keratitis models. Acta Ophthalmol 88: e120–125. 20456251 10.1111/j.1755-3768.2010.01897.x PMC4739651

[60] Slater H, Crow M, Everson L, Salmond GP (2003) Phosphate availability regulates biosynthesis of two antibiotics, prodigiosin and carbapenem, in *Serratia* via both quorum-sensing-dependent and -independent pathways. Mol Microbiol 47: 303–320. 12519208 10.1046/j.1365-2958.2003.03295.x

[61] Miller JH (1972) Experiments in molecular genetics. Cold Spring Harbor, NY: Cold Spring Harbor Laboratory Press.

[62] Marone M, Mozzetti S, De Ritis D, Pierelli L, Scambia G (2001) Semiquantitative RT-PCR analysis to assess the expression levels of multiple transcripts from the same sample. Biol Proced Online 3: 19–25. 12734582 10.1251/bpo20 PMC145543

[63] Stella NA, Fender JE, Lahr RM, Kalivoda EJ, Shanks RM (2012) The LysR transcription factor, HexS, is required for glucose inhibition of prodigiosin production by *Serratia marcescens* Advances in Microbiology *in press*. 10.4236/aim.2012.24065 PMC3865871 24358451

[64] Morowitz MJ, Denef VJ, Costello EK, Thomas BC, Poroyko V, et al (2011) Strain-resolved community genomic analysis of gut microbial colonization in a premature infant. Proc Natl Acad Sci U S A 108: 1128–1133. 21191099 10.1073/pnas.1010992108 PMC3024690

[65] Liaw SJ, Lai HC, Ho SW, Luh KT, Wang WB (2003) Role of RsmA in the regulation of swarming motility and virulence factor expression in *Proteus mirabilis* . J Med Microbiol 52: 19–28. 12488561 10.1099/jmm.0.05024-0

[66] Reimmann C, Ginet N, Michel L, Keel C, Michaux P, et al (2002) Genetically programmed autoinducer destruction reduces virulence gene expression and swarming motility in *Pseudomonas aeruginosa* PAO1. Microbiology 148: 923–932. 11932439 10.1099/00221287-148-4-923

[67] Lai HC, Soo PC, Wei JR, Yi WC, Liaw SJ, et al (2005) The RssAB two-component signal transduction system in *Serratia marcescens* regulates swarming motility and cell envelope architecture in response to exogenous saturated fatty acids. J Bacteriol 187: 3407–3414. 15866926 10.1128/JB.187.10.3407-3414.2005 PMC1112010

[68] Shanks RMQ, Stella NA, Fender JE (2012) Mutation of *crp* mediates *Serratia marcescens* serralysin and global secreted protein production. Res Microbiol *in press*. 10.1016/j.resmic.2012.10.006 PMC3534799 23072819

[69] Fender JE, Bender CM, Stella NA, Lahr RM, Kalivoda EJ, et al (2012) *Serratia marcescens* quinoprotein glucose dehydrogenase activity mediates medium acidification and inhibition of prodigiosin production by glucose. Appl Environ Microbiol 78: 6225–6235. 22752173 10.1128/AEM.01778-12 PMC3416624

[70] Matsuyama T, Murakami T, Fujita M, Fujita S, Yano I (1986) Extracellular vesicle formation and biosurfactant production by *Serratia marcescens* . Journal of General Microbiology 132: 865–875.

[71] Miller VL, Mekalanos JJ (1988) A novel suicide vector and its use in construction of insertion mutations: osmoregulation of outer membrane proteins and virulence determinants in *Vibrio cholerae* requires *toxR* . J Bacteriol 170: 2575. 2836362 10.1128/jb.170.6.2575-2583.1988 PMC211174

[72] Kalivoda EJ, Stella NA, O’Dee DM, Nau GJ, Shanks RM (2008) The cyclic AMP-dependent catabolite repression system of *Serratia marcescens* mediates biofilm formation through regulation of type 1 fimbriae. Appl Environ Microbiol 74: 3461–3470. 18424546 10.1128/AEM.02733-07 PMC2423026

[73] Shanks RM, Kadouri DE, MacEachran DP, O’Toole G A (2009) New yeast recombineering tools for bacteria. Plasmid 62: 88–97. 19477196 10.1016/j.plasmid.2009.05.002 PMC2737453

