# Supplementary material for: A Serratia marcescens PigP Homolog Controls Prodigiosin Biosynthesis, Swarming Motility and Hemolysis and Is Regulated by cAMP-CRP and HexS
Source: PLoS One. 2013 Mar 1;8(3):e57634. doi: 10.1371/journal.pone.0057634 (PMC3585978; doi:10.1371/journal.pone.0057634)
Supplement: Figure S1 — Complementation of prodigiosin phenotype conferred by insertional mutation of pigP . A. Photograph of WT (CMS376) or the pigP mutant (CMS836) with the vector (pMQ125) or ppigP (pMQ212) grown on LB agar supplemented with arabinose (4 mM). B. Prodigiosin production by the environmental isolate, CHASM, and isogenic pigP-insertion mutant (CMS2981) being either the empty vector (pMQ132) or ppigP (pMQ221) grown in LB medium. The average of six independent biological replicates is shown. (PDF) [file pone.0057634.s001.pdf]

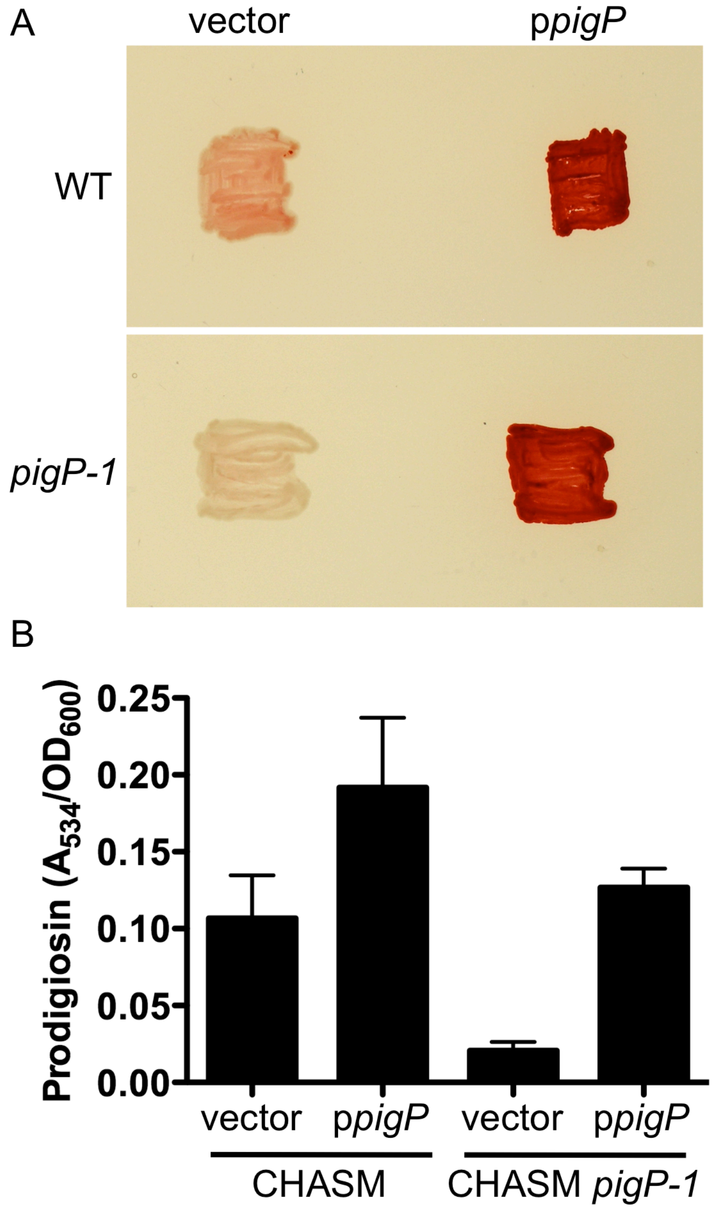

Figure S1. Complementation of prodigiosin phenotype conferred by insertional mutation of *pigP*. A. Photograph of WT (CMS376) or the *pigP* mutant (CMS836) with the vector (pMQ125) or p*pigP* (pMQ212) grown on LB agar supplemented with arabinose (4 mM). B. Prodigiosin production by the environmental isolate, CHASM, and isogenic *pigP*-insertion mutant (CMS2981) being either the empty vector (pMQ132) or p*pigP* (pMQ221) grown in LB medium. The average of six independent biological replicates is shown.
