# Supplementary material for: A Serratia marcescens PigP Homolog Controls Prodigiosin Biosynthesis, Swarming Motility and Hemolysis and Is Regulated by cAMP-CRP and HexS
Source: PLoS One. 2013 Mar 1;8(3):e57634. doi: 10.1371/journal.pone.0057634 (PMC3585978; doi:10.1371/journal.pone.0057634)
Supplement: Figure S2 — Amplification of pigP from S. marcescens isolates. PCR was used to amplify a 138 base pair amplicon from a variety of S. marcescens strains. Db11 and CMS376 served as positive controls, whereas Proteus and Staphylococcus chromosomal DNA served as negative controls. Amplicons were separated on TBE-PAGE gels and stained with ethidium bromide. (PDF) [file pone.0057634.s002.pdf]

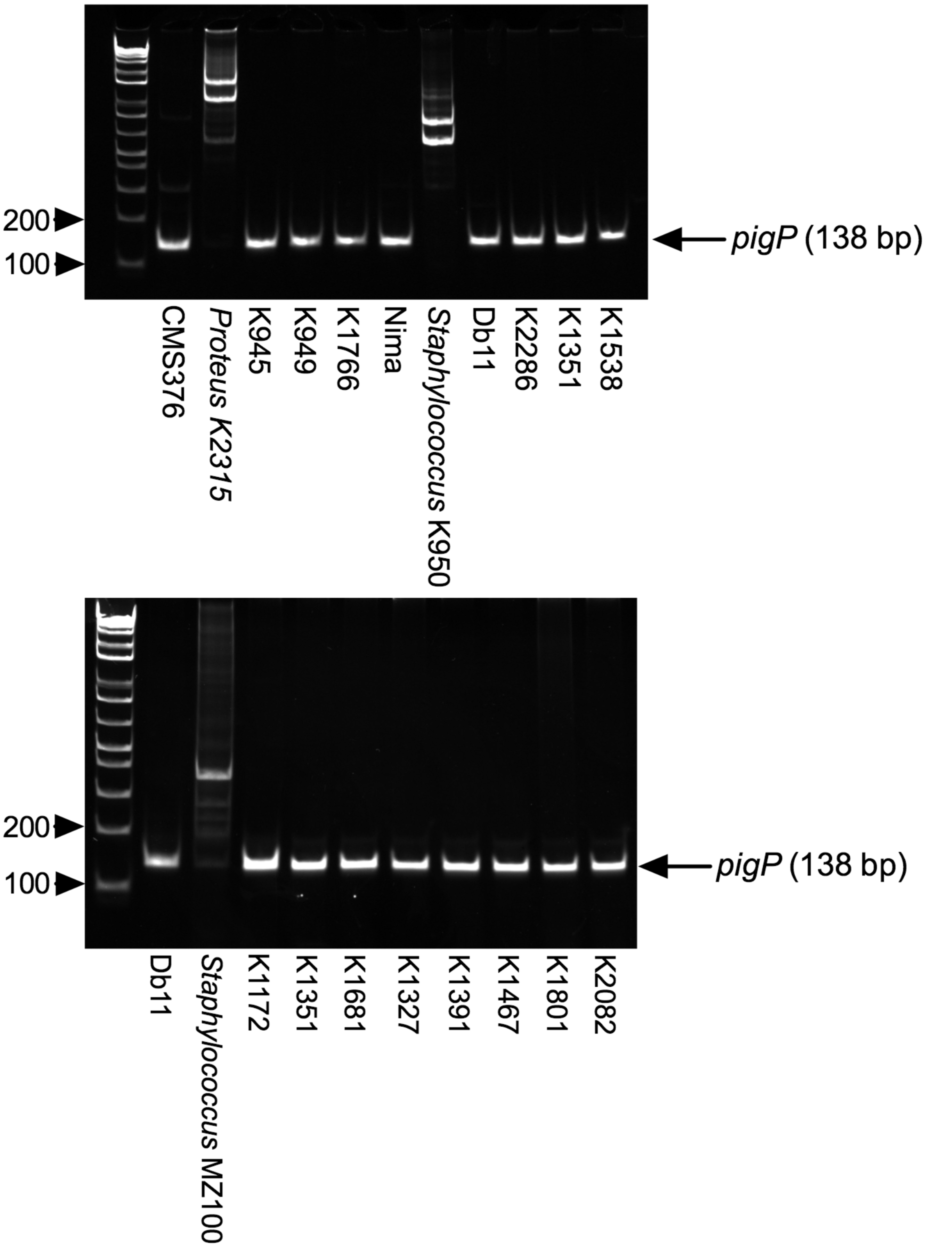

Figure S2. Amplification of *pigP* from *S. marcescens* isolates. PCR was used to amplify a 138 base pair amplicon from a variety of *S. marcescens* strains. Db11 and CMS376 served as positive controls, whereas *Proteus* and *Staphylococcus* chromosomal DNA served as negative controls. Amplicons were separated on TBE-PAGE gels and stained with ethidium bromide.
