# Supplementary material for: A Serratia marcescens PigP Homolog Controls Prodigiosin Biosynthesis, Swarming Motility and Hemolysis and Is Regulated by cAMP-CRP and HexS
Source: PLoS One. 2013 Mar 1;8(3):e57634. doi: 10.1371/journal.pone.0057634 (PMC3585978; doi:10.1371/journal.pone.0057634)
Supplement: Figure S3 — EMSA analysis of His9-PigP and MBP-HexS with promoters of interest. A) His9-PigP exhibited a gel shift of the pigP promoter but not the swrW or hexS promoters. B) MPB-HexS retarded migration of pigA and swrW promoters (positive controls), but not the pswP promoter (negative control) or the hexS promoter. MBP alone did not induce a gel shift of any promoter. Excess unlabeled promoter DNA could compete successfully for MPB-HexS binding to pigA and swrW promoters. Biotin-labeled promoters (Label. Promoter) were used at 2 ng per reaction and unlabeled promoters (Unlab promoter) were used at 500 ng per reaction. (PDF) [file pone.0057634.s003.pdf]

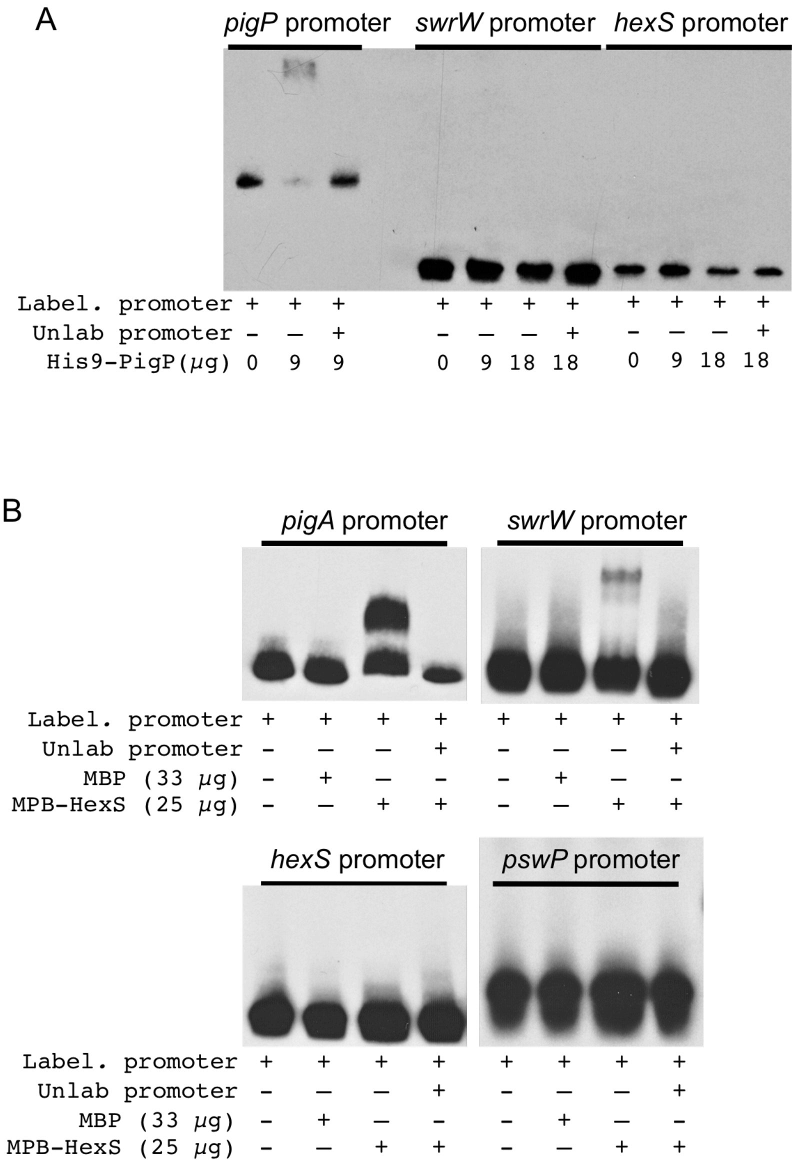

Figure S3. EMSA analysis of His9-PigP and MBP-HexS with promoters of interest. A) His9-PigP exhibited a gel shift of the *pigP* promoter but not the *swrW* or *hexS* promoters. B) MPB-HexS retarded migration of *pigA* and *swrW* promoters (positive controls), but not the *pswP* promoter (negative control) or the *hexS* promoter. MBP alone did not induce a gel shift of any promoter. Excess unlabeled promoter DNA could compete successfully for MPB-HexS binding to *pigA* and *swrW* promoters. Biotin-labeled promoters (Label. Promoter) were used at 2 ng per reaction and unlabeled promoters (Unlab promoter) were used at 500 ng per reaction.
