# Supplementary material for: A Serratia marcescens PigP Homolog Controls Prodigiosin Biosynthesis, Swarming Motility and Hemolysis and Is Regulated by cAMP-CRP and HexS
Source: PLoS One. 2013 Mar 1;8(3):e57634. doi: 10.1371/journal.pone.0057634 (PMC3585978; doi:10.1371/journal.pone.0057634)
Supplement: Figure S4 — Analysis of serratamolide from S. marcescens culture supernatants. LC-MS was used to measure serratamolide levels in culture supernatants from the WT (CMS376) the ΔpigP strain (CMS1713) and the negative control swrW mutant (CMS635). Purified serratamolide was used as a positive control. The serratamolide peaks are boxed in red the scale of the trace is shown on the left hand side. A representative experiment is shown. (PDF) [file pone.0057634.s004.pdf]

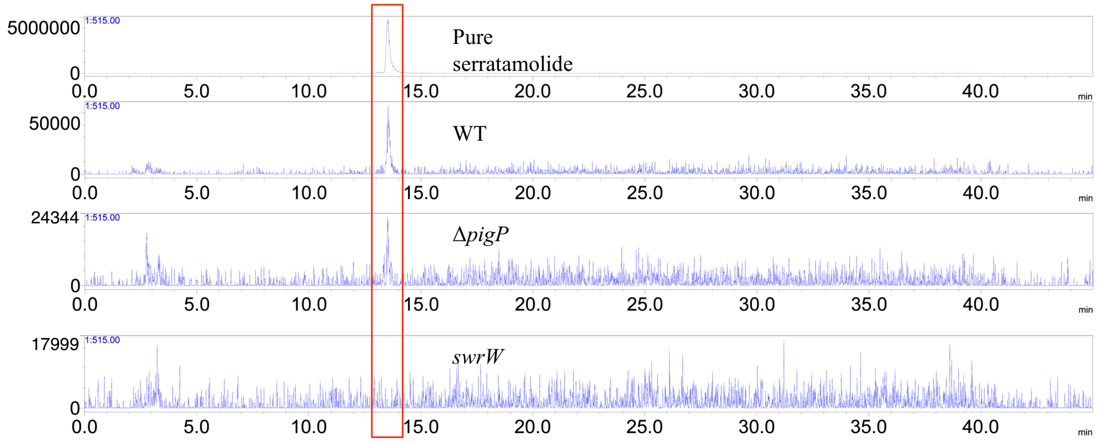

Figure S4. Analysis of serratamolide from *S. marcescens* culture supernatants. LC-MS was used to measure serratamolide levels in culture supernatants from the WT (CMS376) the Δ*pigP* strain (CMS1713) and the negative control *swrW* mutant (CMS635). Purified serratamolide was used as a positive control. The serratamolide peaks are boxed in red the scale of the trace is shown on the left hand side. A representative experiment is shown.
