# Supplementary material for: A Serratia marcescens PigP Homolog Controls Prodigiosin Biosynthesis, Swarming Motility and Hemolysis and Is Regulated by cAMP-CRP and HexS
Source: PLoS One. 2013 Mar 1;8(3):e57634. doi: 10.1371/journal.pone.0057634 (PMC3585978; doi:10.1371/journal.pone.0057634)
Supplement: Table S1 — Primers used in this study. (DOCX) [file pone.0057634.s005.docx]

Table S1. Primers used in this study

Primer number DNA Sequence^a^

736 tgcaggtcgacGGAGGGAAACAATGAATATTCG

737 cccggggatccGCTGTCGAGTTGCGCCAGC

1230 CACAGCGCAAAATCAACATC

1231 CGTTGTTCACTCTTCCGTCA

1238 acgacggccagtgccaagcttgcatgcctgcaggtcgactCACAGCGCAAAATCAACATC

1239 atgttgtgtggaattgtgagcggataacaatttcacacCCTTTTGATGATGATGACCTCG

1337 gttgggtaacgccagggttttcccagtcacgacgttgtaCGCGCACGTCAATGGCCAGC

1338 taacaatttcacacaggaaacagctatgaccatgatCCTCGATCTGCTAAGAACCTTTG

1346 GCTATTACGCCAGCTGGCGAAAGG

1444 cccagtcacgacgttgtaaaacgacgggatctatcatTGGCTGTCCTTACAAACATTACG

1445 aatagaccgagatagggttgagtgttgttccagtttgGGAGTGTTTGACGCTCGAAAAGG

1479 cgacggccagtgccaagcttgcatgcctgcaCGAAATATCAGAAGATCTGAGC

1480 taacaatttcacacaggaaacagctatgaccatgaGTAGGTCTCGCTGTACTCCCAC

1483 tgtatcaggctgaaaatcttctctcatccgccaaagtCCATTTATAACCTTTCTCCAACG

1484 actctctactgtttctccatacccgtaggaggaaaaaaATGAAAACCACCAACGCACAGC

1645 cggataacaatttcacacaggaaacagctaggacagccaATGAAAACCACCAACGCACAG

1646 ttctgttttatcagaccgcttctgcgttctgatcgTTATTATAACCTTTCTCCAACGATG

1665 CGGAATTCAGTATCGAGCGCATTCATGCC

1671 ATTCCTAATGCAGCGCAACTC

1672 TCGGCTACGAGCAGATTTTTG

1673 ATCGCGCTAAATGCTAAAAA

1675 GCTTGAATACGCTGGACAGT

1713 CGACGTCGCTAATATTCATTTTTTCCTCCGG

1851 acgggatctatcattgtttccctccattccatCCCGACAGACTATGTTTAAAAACATACG

1852 gctggcacgacaggtttcccgactggaaagcgCGATAAATCGTTTTGAGATAATTTTAGC

1883 CCTTATAAATCAAAAGAATAGACCGAGATAGG

2093 agaccgcttctgcgttctgatttaatctgtatcaggaTTATAACCTTTCTCCAACGATGT

2094 aaagaatggttcaccaccatcaccaccatcaccaccatAAAACCACCAACGCACAGCGCA

2638 AACTGGAGGAAGGTGGGGAT

2639 AGGAGGTGATCCAACCGCA

2701 CGAGGTCATCATCATCAAAAGG

2702 GCTCAGATCTTCTGATATTTCG

2705 GCTGGGACATCATCAAAACC

2706 CGATTTGGATTCGATCAGGTTGGAAG

2737 CCACTCCGTTATCGTTCACGCTCC

2738 CACGGTCGTCAGGGAATAAGC

2781 CCCGCGTTCTATAAGCACC

2782 GCTCTAATCGCTGCATTTGTTG

2911 GGAGCGAACTGACCTTCAAC

2912 CTGTTCCAGACGCAGTTTCA

2917 CCGTGTGGCTAGACCAATCT

2918 GAACGAAGGTGTGTTTCTGCC

2926 GATATTGCGGATAAAAGTGGGCA

2928 GGAGCGATTTCGAAAAGACC

^a.^ Upper case letter direct amplification of a specific DNA-sequence, lowercase nucleotides directs recombination using yeast recombineering, underlined nucleotides indicate an engineered restriction site.
